# EGR4 transcriptionally upregulates GDF15 to promote gastric cancer metastasis

**DOI:** 10.1038/s41419-025-08095-w

**Published:** 2025-11-07

**Authors:** Weiwei Liu, Yanyan Li, Lixin Liang, Lisheng Zheng, Rui Zeng, Congcong Zhang, Zhihao Lin, Wanying Feng, Qingling Zhang

**Affiliations:** 1 Department of Pathology, Guangdong Provincial People’s Hospital (Guangdong Academy of Medical Sciences), Southern Medical University, Guangzhou, Guangdong China; 2 Guangdong Provincial Key Laboratory of Artificial Intelligence in Medical Image Analysis and Application, Guangdong Provincial People’s Hospital, Guangdong Academy of Medical Sciences, Guangzhou, China; 3 Department of Oral Pathology, Guangdong Provincial Key Laboratory of Stomatology, Hospital of Stomatology, Sun Yat-sen University, Guangzhou, People’s Republic of China

**Keywords:** Gastric cancer, Tumour immunology, Oncogenes

## Abstract

Gastric cancer (GC) metastasis remains a major cause of poor prognosis, yet its molecular drivers are poorly understood. Here, we integrated single-cell RNA sequencing (scRNA-seq) of primary tumors and matched metastatic lymph nodes from six GC patients to identify a metastatic epithelial subpopulation characterized by EGR4 overexpression. Kaplan-Meier analysis revealed that high EGR4 expression correlated with reduced survival in GC patients. Mechanistically, chromatin immunoprecipitation sequencing (ChIP-seq) and luciferase assays demonstrated that EGR4 directly bound to the GDF15 promoter, driving its transcriptional activation. Functional studies showed that EGR4 promoted migration and metastasis via GDF15-mediated ErbB3/ErbB1 hetero-dimerization, which activated PI3K/AKT and MAPK/ERK pathways. Furthermore, CellChat analysis identified robust interactions between EGR4^+^ GC cells and cancer-associated fibroblasts (CAFs), particularly extracellular matrix (ECM)-remodeling eCAFs. Secreted GDF15 induced CAF activation through TGF-β receptor signaling, creating a pro-metastatic niche. Collectively, our study establishes the EGR4/GDF15 axis as a critical driver of GC metastasis, offering possible therapeutic targets for intervention.

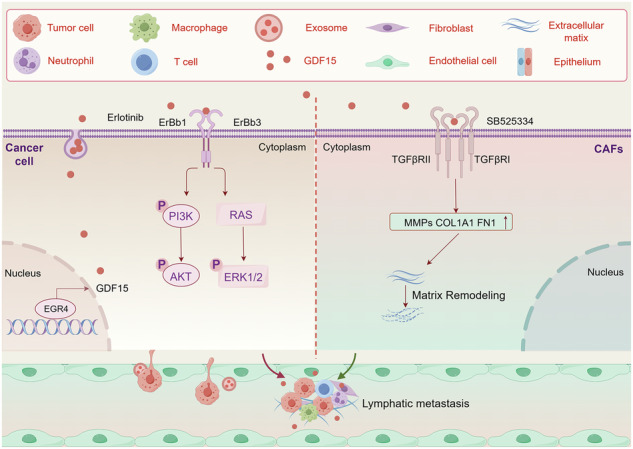

## Introduction

Gastric cancer (GC) is a common malignancy worldwide, with approximately 1 million newly diagnosed cases each year [1, 2]. Despite advancements in diagnosis and treatment in the past decades, the prognosis for advanced GC remains poor, with an overall survival rate of only around 40% [3–5]. Metastasis is an important cause of treatment failure in GC [6]. However, the mechanisms underlying GC metastasis and its biomarker remain unclear.

Single-cell RNA sequencing (scRNA-seq) is a powerful tool providing expression profiles of human cancers and tumor microenvironment (TME) cells at single-cell resolution, enabling to identify and characterize specific sub-clusters with unique biological functions, which has been widely adopted in cancer research to explore the TME and tumor cell evolution [7–9]. Lymphatic metastasis is the most common form of GC metastasis, occurring in both early and advanced GC, affecting its prognosis and treatment decision [10–12]. Therefore, we analyzed scRNA-seq data of GC tissues and paired metastatic lymph nodes to investigate genes involved in GC metastasis, and identified the enrichment of EGR4^+^ cancer cells associated with poor prognosis in metastases.

EGR4, one of the early growth response (EGR) transcription factor family, plays crucial roles in embryonic development, nervous system development, and adult physiology [13–15]. Functioning as a transcription factor, EGR4 regulates the expression of various targeted genes and plays diverse roles in various cancers [16, 17]. It promotes cell growth through transcriptional pathways in small cell lung cancer [18]. In non-small cell lung cancer, EGR4 promotes tumor growth through interacting with long non-coding RNA ZNF205-AS1 in a positive feedback manner [19]. However, downregulation of EGR4 inhibits cholangiocarcinoma tumor cell growth [20]. Up to now, the role of EGR4 in GC metastasis and its mechanism remain unclear.

In this study, we found that EGR4^+^ cancer cells play a pivotal role in GC metastasis by upregulating GDF15, which can bind ErbB3 to enhance ErbB3/ErbB1 hetero-dimerization and the activation of ErbB1 and its downstream MAPK/ERK and PI3K/AKT signaling, while induce CAFs activation via the TGF-βR pathway to in turn further promote GC metastasis. Our study highlights the critical role of EGR4/GDF15 axis in GC metastasis, underscores its potential as a prognosis biomarker and therapeutic target of GC.

## Materials and Methods

### Patient and sample collection

Human GC surgical samples (12 cases, with patient information shown in Supplementary Table 1) were collected from Guangdong Provincial People’s Hospital and paraffin-embedded specimens (tissue micro array, TMA) were purchased from Taize Biotechnology Co, with a total of 72 cases and the patient information shown in supplementary Table 2. This study was approved by the hospital’s ethics committee (ID: KY2025-458-01) and all participants provided written informed consent.

### Cell lines and culture

Human GC cell lines HGC27 and MKN45 were purchased from the ATCC cell bank, and human GC tissue-derived fibroblasts were purchased from Shenzhen Youli Biotechnology Co. HGC27 cells were cultured in DMEM high-glucose medium (Procell, Cat#PM150110) with 10% fetal bovine serum (FBS; Sigma-Aldrich, Cat#F0193), while MKN45 cells were cultured in RPMI-1640 (Procell, Cat#PM150110) complete medium with 10% FBS. Fibroblasts were cultured in iCell primary fibroblast-specific medium (Procell, Cat#CM-h206). All cells were maintained in T25 flasks at 37 °C with 5% CO_2_ in an incubator with the medium replaced every two days.

### Western blot analysis

Cells were first lysed in RIPA buffer (ECOTOP SCIENTIFIC,Cat#ES-8148-100) containing protease and phosphatase inhibitors (NCM,Cat#P003) on ice for 30 min. And then, cell lysates were clarified through centrifugation at 12,000 g at 4 °C for 20 min and equal mass of proteins (20 µg) were separated by SDS-PAGE, following by transferred to PVDF membranes (Millipore, USA) through Bio-Rad’s wet transfer system. Membranes were first blocked in TBST with 5% BSA and then incubated with appointed primary antibodies. After washing thrice with TBST, the membranes were then incubated with corresponding HRP-conjugated secondary antibodies at room temperature for 1 h. Finally, the proteins were visualized using GelView 6000 Plus (BLT Photon Technology, China) according to the manufacturer’s instructions. All experiments were repeated three times with the antibody information and dilution multiple provided in Supplementary Table 3. Relative protein expression levels were quantified using ImageJ software version 1.54 p.

### Immunohistochemistry

Tissues were fixed and embedded in paraffin, and 3µm-thick sections were prepared. After de-paraffinization, antigen retrieval using EDTA antigen retrieval solution (biosharp, Cat#BL617A) and endogenous peroxidase activity quenching with hydrogen peroxide, the sections were then washed three times with PBS and the non-specific binding sites were blocked with 5% goat serum at room temperature for 1 h. Thereafter, the sections were incubated with primary antibodies at 4 °C overnight, followed by three washes with PBS and then incubated with HRP-conjugated corresponding secondary antibodies at room temperature for 1 h. After three washes with PBS, the sections were developed using a DAB kit (ZSGB-BIO, Cat#ZLI-9019) for 5–10 minuntil the desired color was observed. Afterwards, the nuclei were counterstained by hematoxylin, and then sections were dehydrated, cleared and mounted. Finally, images were captured using a microscope and analyzed with ImageJ software. The antibody information and dilution multiple are provided in Supplementary Table 3.

### Immunofluorescence analysis

Multiplex staining was performed using the PANO 7-plex IHC kit (CAT 0004100100, Panovue, Beijing, China). Sections were sequentially incubated with appointed primary antibodies, HRP-conjugated corresponding secondary antibodies and tyramide signal amplification (TSA) substrates. After each round of TSA treatment, sections were subjected to microwave heat treatment. Nuclei were stained with DAPI after all antigens were labeled. The antibody information and dilution multiple are provided in Supplementary Table 3.

### RT-qPCR analysis

Total RNA was first extracted using the TRIzol reagent (AG,Cat#AG21102), and then measured with NanoDrop to detect RNA concentration and purity. RNA was reverse-transcribed using a reverse transcription kit, and SYBR Green PCR Master Mix (AG,Cat#AG11718) was used for amplification on LightCycler 480 Fast Real-Time PCR system (Roche). The reaction consisted of 40 cycles of 95 °C for 10 s and then 60 °C for 30 s. GAPDH was used as an internal control, and gene expression was analyzed using the 2^-ΔΔCt^ method. The primer sequences are listed in Supplementary Table 4.

### Immunoprecipitation

Cells were washed twice with ice-cold PBS and then lysed in lysis buffer containing protease and phosphatase inhibitors and PMSF (ECOTOP SCIENTIFIC,Cat#ES-8134-10). Cell lysates were incubated on ice for 30 min and centrifuged at 12,000 g at 4 °C for 30 min. Equal lysates were incubated with fresh protein A/G beads (Beyotime,Cat#P2181S) and 1–4 µg of antibody overnight at 4 °C. Unbound proteins were removed by washing thrice with lysis buffer or PBS. After elution, immunoblotting was performed using specific primary antibodies. The antibody details and dilution multiple are provided in Supplementary Table 3.

### Plasmids, siRNA, shRNA and transfection

Wild-type and mutant Flag-EGR4 plasmids were purchased from Guangzhou Hanyi Biotechnology Co. ShRNA and siRNA specially targeting EGR4 and GDF15 were synthesized by Guangzhou Jiyuan Biotechnology Co. Plasmid and siRNA were transfected using Lipofectamine 3000 (Invitrogen, USA). Sequences of siRNA and shRNA are listed in Supplementary Table 5.

### ELISA

The GDF15 concentration was measured with the ELISA kit (HBDY, Cat#HBDY-1643H2). Briefly, the plates were first equilibrated at room temperature for 20 min. Standards and samples were then added to designated wells, followed by HRP-conjugated detection antibody. After incubation at 37 °C for 60 min, the plates were washed thrice, and substrate A and B were then added. After 15 min of incubation at 37 °C in the dark, the reaction was stopped, and absorbance was measured at 450 nm.

### Transwell migration and invasion assay

Cells in 200 µL FBS free medium (1 × 10^5^ cells/mL) were seeded in the upper chamber of a 24-well transwell plate (CORNING,Cat#353097), with the lower chamber containing medium with 10% FBS as a chemoattractant. For invasion assay, the cells were plated in the Matrigel pre-coated upper chamber. After 24–72 h incubation at 37 °C with 5% CO_2_, the cells were fixed with methanol and non-migrated/invaded cells were removed, and migrated/invaded cells were stained with 0.1% crystal violet. Cells in five random fields were counted.

### Subcutaneous tumor formation and lung metastasis model in nude mice

MKN45 cells (1 × 10^6^ cells) were suspended in 100 µL serum-free medium and injected subcutaneously into the flank of four-week-old male BALB/c nude mice (*n* = 5 per group) (purchased from Guangdong Medical Laboratory Animal Center) weighing 15–20 g. Two weeks later, the mice were euthanized and the tumors were harvested for histological analysis. For metastasis model, MKN45 cells (1 × 10^6^ cells) were suspended in 100 µL of serum-free medium and injected into the tail vein of four-week-old male BALB/c nude mice. Mice were monitored weekly for body weight and general health. After five weeks, the mice were euthanized and lung tissues were harvested to assess metastatic nodules. Animals were kept in the SPF animal breeding room of Daoke Biological (Guangzhou, China). All operations were carried out in accordance with relevant guidelines and regulations and were approved by the Animal Ethics Committee (number: KY2025-458-01).

### ChIP-seq and ChIP-qPCR

Fresh cells were cross-linked in 1% formaldehyde, and the reaction was quenched with 125 mM glycine. Chromatin was then sheared to 100–500 bp fragments using sonication. Flag antibody (1–5 µg) was incubated with chromatin fragments overnight at 4 °C and protein A/G beads (Beyotime,Cat#P2181S) (1–5 µg) were then added to incubate for additional 4–6 h at 4 °C. Beads were sequentially washed with low-salt, high-salt, LiCl, and TE buffers, and then DNA was eluted with 1% SDS and 0.1 M NaHCO3. After crosslink reversal at 65 °C overnight, DNA was purified using Qiagen DNA extraction kit. DNA was analyzed by qPCR or sequencing. For ChIP-qPCR assay, DNA was analyzed using SYBR Green on a QuantStudio 5 qPCR system. Experiments were repeated three times, and the primer sequences are listed in Supplementary Table 4. Data were analyzed using two-tailed t-tests, with *P* < 0.05 considered statistically significant.

### Dual-luciferase reporter assay

Wild-type and mutant *GDF15* promoter were synthesized and inserted into the pGL3-basic vector (Promega). The plasmids were co-transfected with Renilla luciferase expression plasmid (Promega) into cells using Lipofectamine 2000. Forty eight hours later, the luciferase activity was then measured using a dual-luciferase reporter kit (TransDetect, Cat#fr201-02-v2), and signals were detected using a Infinite M200 PRO plate reader.

### RNA sequencing

Total RNA was first extracted using the TRIzol reagent (Invitrogen), and the Poly(A) RNA was enriched and fragmented using the magnesium RNA fragmentation module at 94 °C. RNA was then reverse-transcribed into cDNA using SuperScript™ II reverse transcriptase and sequenced on an Illumina Novaseq™ 6000. Transcript expression was analyzed using StringTie and edgeR, and differentially expressed genes (log2 fold change >1 or <−1, *P* < 0.05) were identified. Volcano plots were used to visualize differentially expressed genes, and Gene Ontology (GO) analysis was performed.

### Single-cell RNA-seq data analysis

The single-cell libraries were prepared using the Chromium Single Cell Gene Expression Solution, Chromium Single Cell 5’ Gel Bead, Chip, and Library Kits v2 (10X Genomics) and cells were loaded at a density of 8000–10,000 cells per channel to ensure optimal recovery. Cells were partitioned into gel beads in the Chromium device for mRNA barcoded reverse transcription and cell lysis, followed by amplification, fragmentation, 5’ adapter addition and sample indexing. For cell-cell communication analysis, the R package “CellChat” (v1.4.0) was used to calculate and visualize cell communication networks. The KOBAS 3.0 and GOseq R packages were used for Kyoto Encyclopedia of Genes and Genomes (KEGG) and Gene Ontology (GO) enrichment analysis of differentially expressed genes (DEGs). For regulatory networks analysis, the pySCENIC (v0.12.0) was used, with the Scanpy.AnnData object from epithelial tissue read counts used to calculate transcription factor activity. Cell clusters were used as input, and the AUC matrix was generated using default parameters, with the regulatory specificity scores (RSS) used for predicting for each cell cluster.

### Statistical analysis

GraphPad Prism 8.0 was used for statistical analysis. Differences between groups were assessed using two-tailed unpaired *t*-tests or Mann-Whitney tests according to the results of normal distribution analysis, while differences among three or more groups were analyzed through one-way ANOVA method followed by Tukey’s post hoc test. The survival curves were plotted using the Kaplan-Meier method and compared by the log-rank test. Categorical data were compared using Fisher’s exact test or chi-square test. All experiments, except for those involving mice, were performed in at least three independent biological replicates, with technical replicates for each experiment. *P* < 0.05 was considered statistically significant.

## Results

### Identification of cancer cell clusters enriched in GC metastasis

**Fig. 1:**
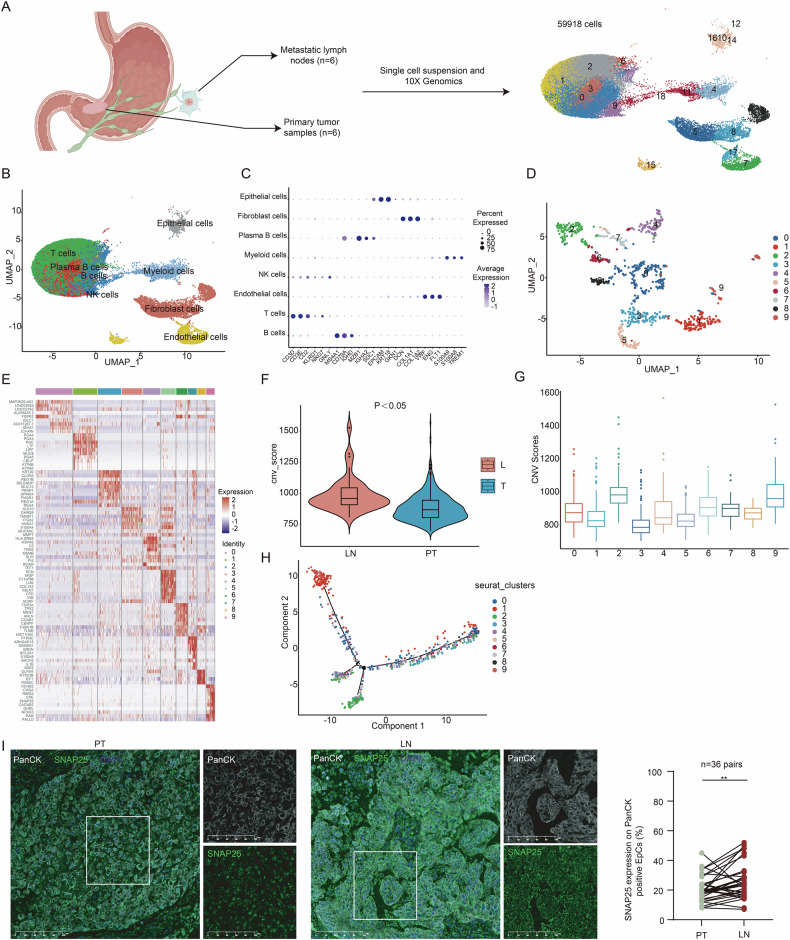
Identification of cancer cell clusters enriched in GC metastasis. **A** Pipeline of the scRNA-seq study design. **B** UMAP visualization of 59,918 cells color-coded by major cell types. **C** Bubble plots showing expression of marker genes across the major cell types. **D** UMAP of epithelial cells revealing 10 distinct clusters. **E** Heatmap of the top marker genes in each epithelial sub-cluster. **F** CNV analysis in epithelial cells from primary tumors and lymph nodes. **G** CNV analysis in epithelial sub-clusters. **H** Trajectory analysis showing two distinct cell fates for epithelial cells. **I** Immunofluorescence staining and quantitative analysis of PanCK^+^ SNAP25^+^ cells in sections of paired primary tumor and lymph node metastasis. For each tissue section, three randomly selected fields of view (FOVs) were analyzed, and the positivity rate of SNAP25 in PanCK-positive epithelial cells was calculated for each FOV, with the average value representing the SNAP25 expression level per sample. **p < 0.01by paired t-test. *p < 0.05; **p < 0.01; ***p < 0.001.

To comprehensively clarify and understand the cell subsets that may play an important role in GC metastasis, we collected six pairs of primary gastric tumors and paired metastatic lymph nodes for scRNA-seq (by 10x Genomics). All lymph node samples were confirmed as metastatic by H&E staining of slides and the confirmation of three pathologists. After filtering out the damaged or dead cells and potential doublets, a single-cell atlas consisting of 59,918 cells was constructed (Fig. 1A). Using uniform manifold approximation and projection (UMAP), we generated a two-dimensional map with 19 clusters across 12 samples (Figure S1A, B). These clusters were annotated into eight cell types, including epithelial cells (EPCAM, KRT18, GKN1 as markers), fibroblasts (DCN, COL1A1, COL1A2 as markers), plasma B cells (MZB1, IGHA2, SDC1 as markers), myeloid cells (S100A9, S100A8, TREM1 as markers), natural killer (NK) cells (KLRD1, NKG7, GNLY as markers), endothelial cells (VWF, ENG, FLT1 as markers), T cells (CD3D, CD3E, CD2 as markers), and B cells (MS4A1, CD79A, IGHD as markers) (Fig. 1C and S1C).

Given that GC cells originate from epithelial cells, unsupervised clustering analysis on the UMAP were performed and the epithelial cells was re-clustered into 10 distinct clusters (Fig. 1D), with the expression of the top cluster-specific genes shown in Fig. 1E. To distinguish the malignant clusters from non-malignant clusters, we assessed the copy number variation (CNV) levels of each epithelial cell cluster, revealing that epithelial cells in the lymph nodes exhibited significantly higher CNV levels than those in primary tumors (Fig. 1F). Among all the 10 epithelial cell clusters, the C3 cluster had lowest CNV levels (Fig. 1G), suggesting that the C3 cluster represented a population of normal gastric epithelial cells, while the other clusters representing malignant epithelial cells [21, 22]. To dissect the evolutionary dynamics of the gastric epithelial lineage, a pseudo-time trajectory analysis for the 10 epithelial cell clusters was performed, generating a four-branch trajectory revealing the progression from non-malignant to malignant and finally metastatic cells (Fig. 1H). The C3 cluster was positioned at the upper right corner in the trajectory curve, indicating as a clear starting point of the trajectory, and the developmental route was determined to start from the initial state and then bifurcate into metastasis-related cell fate 1 or 2. Since C2 cluster and C9 cluster were respectively located at one end of the trajectory curve exhibiting relatively higher CNV levels (Fig. 1G, H), these two sub-clusters were thus suggested to be associated with GC metastasis. Subsequently, Kaplan-Meier survival analysis of the representative genes from C2 cluster and C9 cluster showed that higher expression levels of the representative genes from C2 cluster were related to higher survival rates in GC patients, while higher expression levels of the representative genes from C9 cluster were related to lower survival rates (Figure S2 and S3). SNAP25, a gene specifically enriched in C9 cluster, was selected as a biomarker for C9 cluster. Dual immunofluorescence staining of 36 pairs of metastatic lymph nodes and paired primary gastric tumors revealed that PanCK^+^ SNAP25^+^ expression was higher in the metastatic lymph nodes than that in the primary tumors (Fig. 1I). In summary, these data indicated that the malignant C9 cluster was significantly associated with GC metastasis and attracted more attention for further study.

### EGR4 was upregulated and positively associated with GC metastasis

**Fig. 2:**
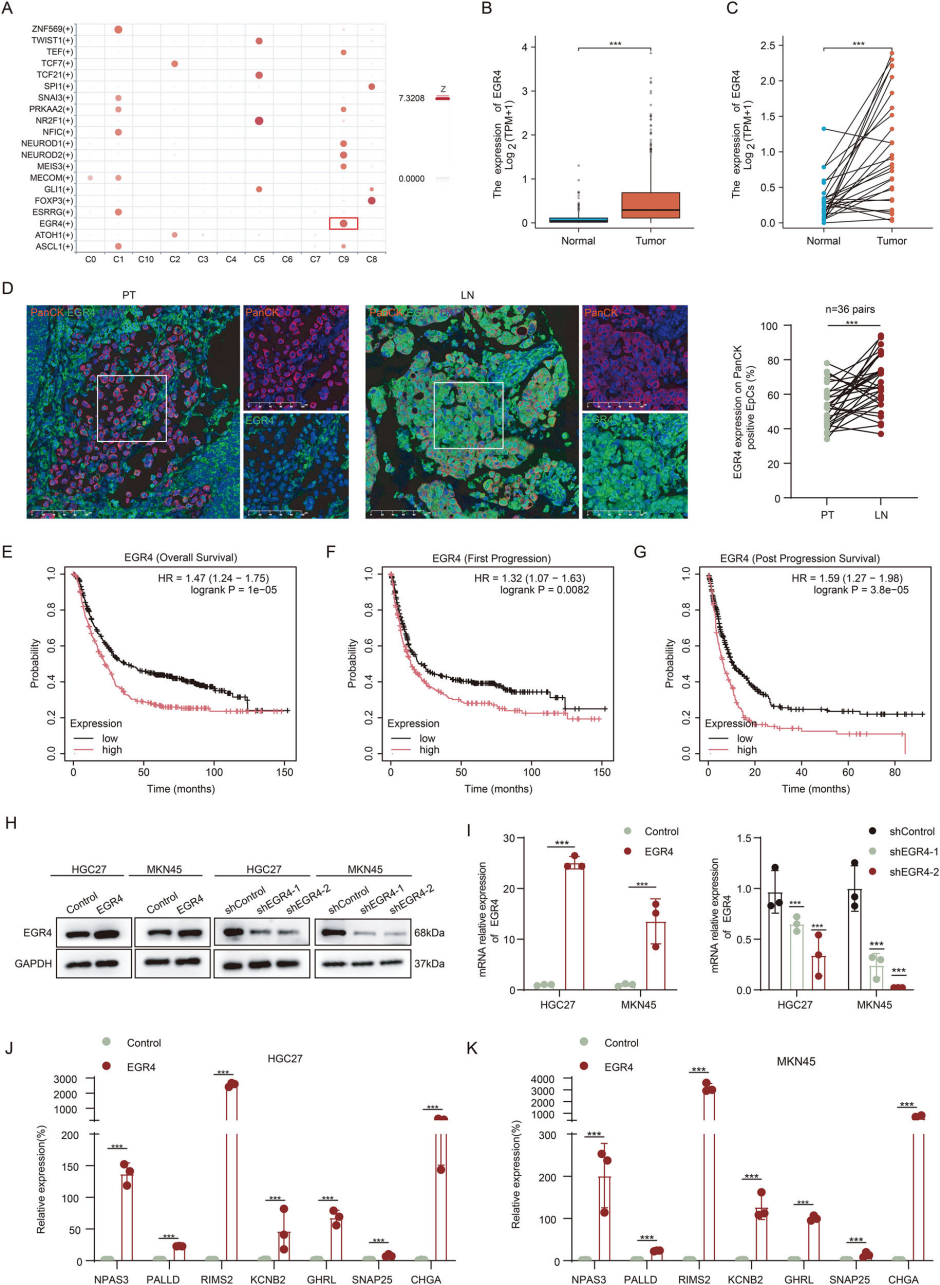
EGR4 was upregulated and positively associated with GC metastasis. **A** Dot plots showing regulon activities in different epithelial cell sub-clusters. **B** *EGR4* mRNA expression in the TCGA-GC dataset. **C** *EGR4* mRNA expression in paired para-cancerous and tumor tissues from GC patients. **D** Immunofluorescence staining and quantitative analysis of PanCK^+^ EGR4^+^cells in sections of paired primary tumor and lymph node metastasis. For each tissue section, three randomly selected FOVs were analyzed, and the positivity rate of EGR4 in PanCK-positive epithelial cells was calculated for each FOV, with the average value representing the EGR4 expression level per sample. ***p < 0.001by paired t-test. **E–G** Kaplan-Meier survival analysis of GC patients based on *EGR4* expression levels. **H**, **I** Western blot and RT-qPCR validation of EGR4 overexpression and knockdown in GC cells. **J**, **K** RT-qPCR analysis of expression of C9 marker genes upon EGR4 over-expression. *p < 0.05, **p < 0.01, ***p < 0.001.

We used the single-cell regulatory network inference and clustering (SCENIC) method to identify key regulators in C9 cluster by linking cis-regulatory sequence information with scRNA-seq data. SCENIC analysis showed that EGR4, NEUROD1 and NEUROD2 showed the highest regulatory activity in C9 the cluster (Fig. 2A). Kaplan-Meier survival analysis revealed that higher NEUROD1 and NEUROD2 levels were associated with higher survival rates in GC patients (Figure S4). However, analysis of EGR4 expression in GC patients through TCGA data sets and paired para-cancerous and tumor tissues showed that it was upregulated in GC samples (Fig. 2B, C), and dual immunofluorescence staining also demonstrated that PanCK^+^ EGR4^+^ expression was higher in the metastatic lymph nodes than that in the primary tumors (Fig. 2D). Furthermore, Kaplan-Meier survival analysis indicated that higher EGR4 expression was associated with lower patient survival rates (Fig. 2E–G). These results indicate that EGR4 can serve as a prognosis biomarker for GC metastasis. Subsequently, we constructed EGR4 overexpression and knockdown GC cell lines using lentivirus (Fig. 2H, I), and found that EGR4 overexpression upregulated the expression of representative genes of the C9 cluster, including NPAS3, PALLD, CHGA, RIMS2, KCNB2, GHRL and SNAP25 (Fig. 2J, K). These data indicate that EGR4 in gastric cancer epithelium may be a key regulator of the C9 cluster; Therefore, we named the C9 cancer cell subpopulation as the EGR4^+^ cancer cell subpopulation.

### EGR4 promoted GC cell migration, invasion and metastasis

**Fig. 3:**
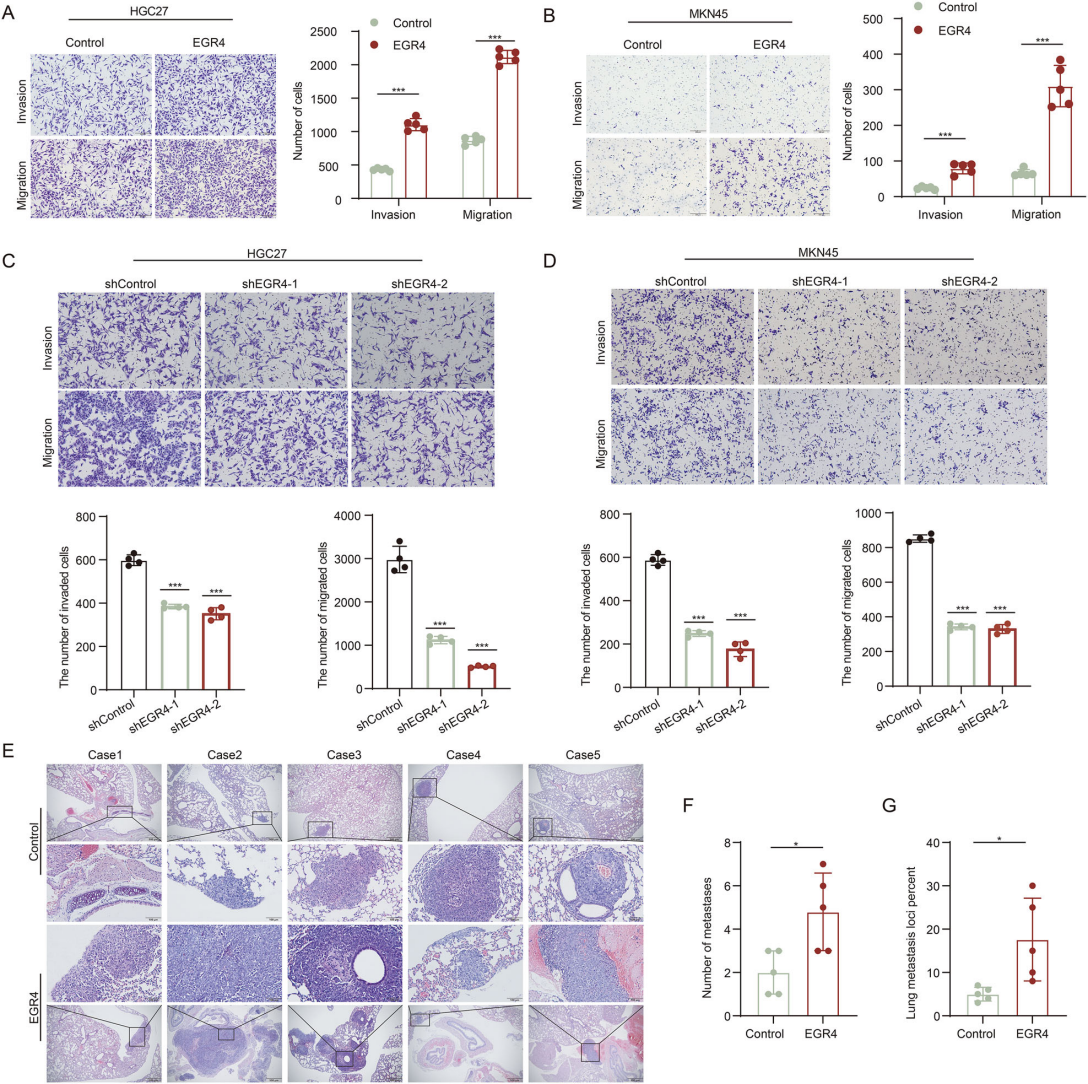
EGR4 promoted GC cell migration, invasion and metastasis. **A**, **B** Transwell assays showed the effect of EGR4 over-expression on migration and invasion of HGC27 and MKN45 cells. **C**, **D** Transwell assays showed the effect of EGR4 knockdown on migration and invasion of HGC27 and MKN45 cells. **E** H&E staining of lung metastatic lesions in the in vivo lung metastasis mice model. **F**, **G** Quantitative analysis of the number of metastases and percentage of metastasis loci in the in vivo lung metastasis mice model. *p < 0.05, **p < 0.01, ***p < 0.001.

To verify the function of EGR4 in GC metastasis, we performed a series of in vitro and in vivo experiments. Transwell migration and invasion assays revealed that EGR4 overexpression dramatically promoted GC cell migration and invasion (Fig. 3A, B), while EGR4 knockdown dramatically inhibited GC cell migration and invasion (Fig. 3C, D). More importantly, in nude mice tail vein-lung metastasis model, we found that EGR4 overexpression significantly enhanced tumor cell colonization in the lungs (Fig. 3E–G). These results confirmed that EGR4 played a pivotal role in promoting GC metastasis.

### EGR4 transcriptionally upregulated GDF15 expression in GC cells

**Fig. 4:**
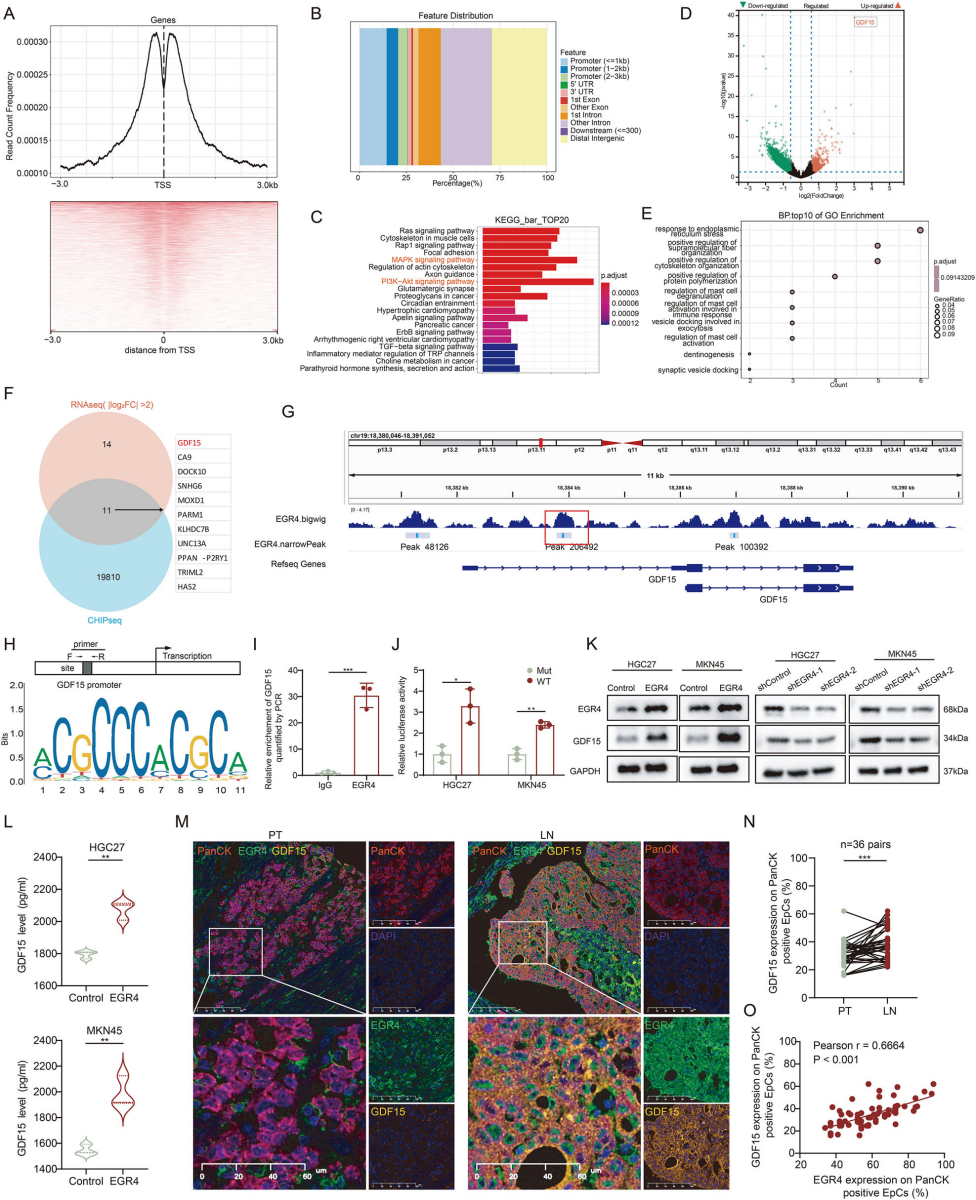
EGR4 transcriptionally upregulated GDF15 expression in GC cells. **A**, **B** Distribution of EGR4 binding peaks near transcription start sites (TSS) and gene structures. **C** KEGG pathway enrichment analysis of the EGR4 target genes. **D** Volcano plot of EGR4-regulated genes by RNA-seq. **E** GO analysis of EGR4-regulated genes by RNA-seq. **F** Venn diagram integrating ChIP-seq and RNA-seq data to identify direct target and effector genes of EGR4, including *GDF15*. **G** Diagram showing the distribution of EGR4 binding peaks near TSS of *GDF15* gene by ChIP-seq. **H** Diagram showing the conserved recognition sequence of EGR4 in *GDF15* promoter region. **I** ChIP-qPCR confirming EGR4 binding to the GDF15 promoter. **J** Luciferase reporter gene assay confirming EGR4 activating the transcriptional activity of GDF15 promoter. **K**, **L** Western blot and ELISA assays confirming the upregulation of GDF15 expression by EGR4. **M** Immunofluorescence staining of EGR4 and GDF15 expression in PanCK^+^ cells in paired primary tumor and lymph node metastasis sections from 36 GC patients. **N** Quantitative analysis of the GDF15 expression in PanCK^+^ cells. For each tissue section, three randomly selected FOVs were analyzed, and the positivity rate of GDF15 in PanCK-positive epithelial cells was calculated for each FOV, with the average value representing the GDF15 expression level per sample. ***p < 0.001by paired t-test. **O** Correlation between EGR4 and GDF15 expression in PanCK^+^ cells. For each tissue section, three randomly FOVs were analyzed, and the positivity rate of EGR4 or GDF15 in PanCK-positive epithelial cells was calculated for each FOV, with the average value representing the EGR4 or GDF15 expression level per sample, respectively. ***p < 0.001by Pearson’s correlation analysis. *p < 0.05, **p < 0.01; ***p < 0.001.

We hypothesized that the potential mechanism by which EGR4 promoted GC metastasis may depend on its direct gene targets as a transcription factor. To identify the direct gene targets of EGR4, we performed ChIP-seq and RNA-seq. Notably, EGR4 binding sites were significantly enriched around transcriptional start sites (TSS, −3 kb to +3 kb) (Fig. 4A). Genomic annotation of peaks revealed their distribution across various genomic elements (Fig. 4B). Moreover, Kyoto Encyclopedia of Genes and Genomes (KEGG) pathway analysis identified significant enrichment in ErbB signaling, Ras signaling, MAPK signaling and PI3K-Akt signaling pathways (Fig. 4C). On the other hand, RNA-seq analysis of EGR4-overexpressing cells revealed the differentially expressed genes (Fig. 4D), with Gene Ontology (GO) analysis highlighting immune response and extracellular secretion pathways (Fig. 4E). By cross-referencing the ChIP-seq and RNA-seq data, we identified 11 overlapping genes, including GDF15, which showed the highest upregulation in the immune response and extracellular secretion pathway (Fig. 4F). Growth/differentiation factor 15 (GDF15), also known as macrophage inhibitory cytokine-1 (MIC1) and nonsteroidal anti-inflammatory drug-activated gene-1 (NAG-1), is considered as a cachexia factor and has been used as a biomarker for tumor diagnosis and prognosis [23–29]. Genomic distribution of the EGR4 enriched peaks revealed their location on *GDF15* promoter region containing conserved recognition sequence of EGR4 (Fig. 4G, H). ChIP-qPCR experiments demonstrated significant enrichment of DNA fragments at the *GDF15* promoter region in EGR4 antibody-immunoprecipitated DNA-protein complexes (Fig. 4I). We performed a promoter luciferase reporter assay to investigate whether EGR4 binding to the *GDF15* promoter region enhanced *GDF15* transcriptional activity, and the results showed that the mutant (MUT) luciferase reporter with a mutated EGR4 binding site exhibited dramatically reduced luciferase activity compared to the wild-type (WT) reporter (Fig. 4J), indicating that EGR4 can activate GDF15 transcription by direct binding to the motif. Western blot and ELISA assay further validated that EGR4 upregulated GDF15 expression and secretion (Fig. 4K, L). In addition, immunofluorescence staining showed higher GDF15 expression on PanCK^+^ EGR4^+^ cells in the metastatic lymph nodes than that in primary tumors, with a positive correlation between GDF15 and EGR4 expression (Fig. 4M, N). These results confirmed that GDF15 was a direct target of EGR4 in GC cells.

### EGR4 activated ErbB3/ErbB1 and downstream MAPK/ERK and PI3K/AKT signaling via GDF15

**Fig. 5:**
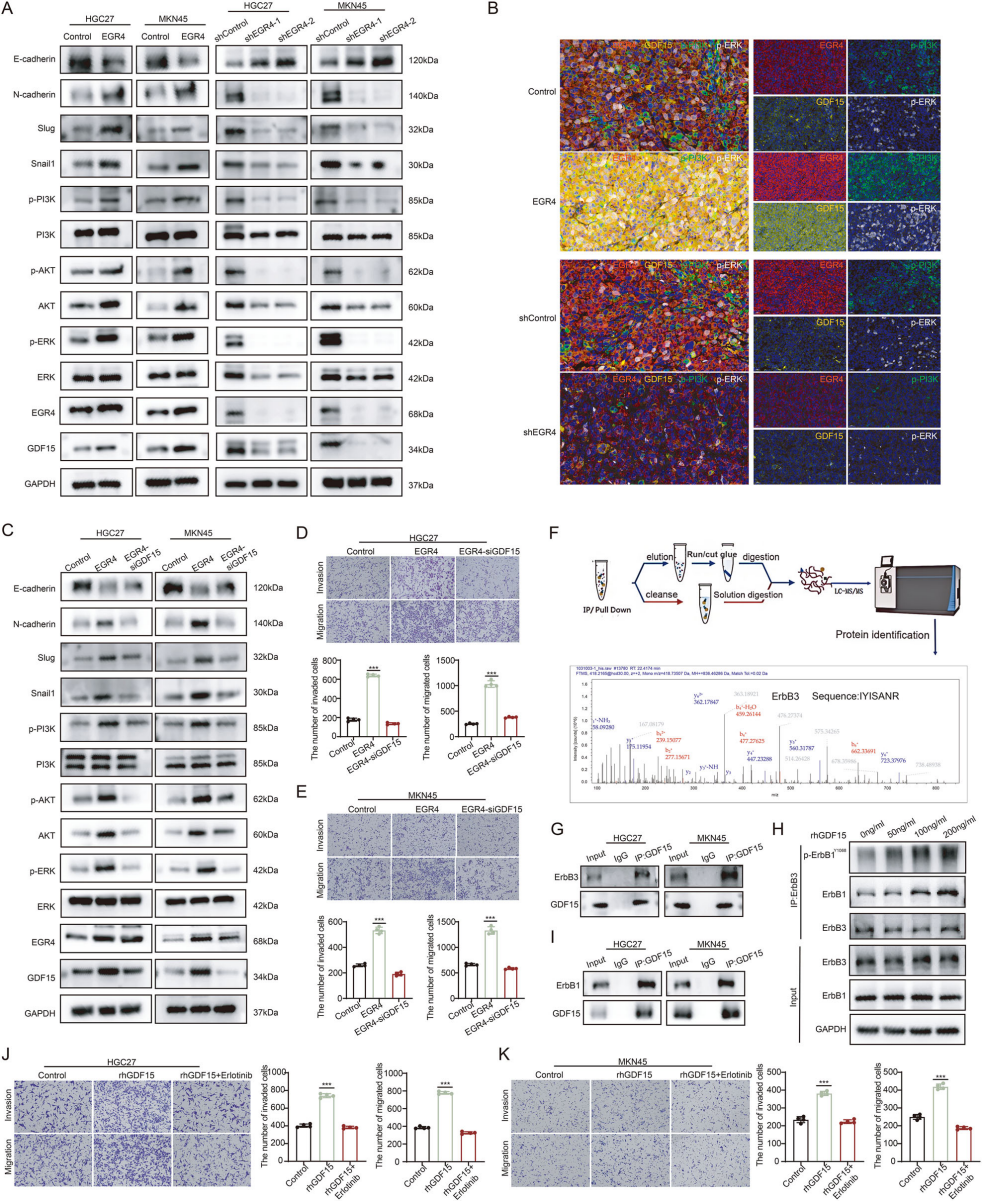
EGR4 activated ErbB3/ErbB1 signaling and downstream MAPK/ERK and PI3K/AKT via GDF15. **A** Western blot showing the effect of EGR4 on the expression of the proteins of MAPK/ERK and PI3K/AKT signaling and EMT markers in GC cells. **B** The immunofluorescence images show the co-localization of EGR4, GDF15, p-PI3K and p-ERK in the nude mouse model. **C** Western blot showing the effect of GDF15 knockdown on the expression of the proteins of MAPK/ERK and PI3K/AKT signaling and EMT markers in EGR4-overexpressing GC cells. **D**, **E** Transwell assays showing the effect of GDF15 knockdown on the migration and invasion of EGR4-overexpressing GC cells. **F** Schematic diagram of Mass spectrometry analysis of GDF15 interactors and the representative peptide mass spectrometry peaks of ErbB3 in GDF15 co-IP complex. **G** Co-IP/western blot showing the binding of GDF15 and ErbB3. **H** Co-IP/western blot showing the effect of rhGDF15 treatment on the binding of ErbB3 with ErbB1 and the phosphorylation of ErbB1. **I** Co-IP/western blot showing the binding of GDF15 and ErbB1. **J**, **K** Transwell assay showing the inhibitory effect of Erlotinib (10 µmol/l) on the migration and invasion of rhGDF15 (200 ng/ml) stimulated GC cells. *p < 0.05, **p < 0.01, ***p < 0.001.

Based on GO analysis of ChIP-seq data, we found that EGR4 was significantly associated with pathways such as ErbB signaling and Ras signaling, MAPK signaling, PI3K-Akt signaling, which could be downstream of ErbB signaling [30]. Therefore, we further investigated whether EGR4 activated the MAPK/ERK and PI3K/AKT signaling pathways in GC cells through GDF15. Western blot confirmed that EGR4 activated the MAPK/ERK and PI3K/AKT signaling pathways and Epithelial-Mesenchymal Transition (EMT), a key characteristic of metastatic cells (Fig. 5A). Immunostaining of tissue sections from mice also showed that overexpression of EGR4 upregulated the level of GDF15, p-PI3K and p-ERK, whereas knocking down EGR4 decreased the level of GDF15, p-PI3K and p-ERK (Fig. 5B and Figure S5). Besides, we transfected GC cells with siRNA targeting GDF15, and the results indicated that knocking down GDF15 would impair the activation of the PI3K/AKT and MAPK/ERK signaling pathways and EMT in EGR4-overexpressing gGC cells (Fig. 5C). Additionally, transwell assays revealed that knockdown of GDF15 significantly reduced migration and invasion of EGR4-overexpressing GC cells (Fig. 5D, E). These results indicate that EGR4 may activate the MAPK/ERK and PI3K/AKT signaling pathways through GDF15, thereby promoting GC cell motility.

To explore the specific mechanism by which GDF15 activated the MAPK/ERK and PI3K/AKT pathways, we performed anti-GDF15 co-IP/mass spectrometry and found that GDF15 could bind to ErbB3 (Fig. 5F), which was validated by co-IP/WB (Fig. 5G). Studies have shown that ErbB3 can form heterodimers with ErbB1 and activate downstream MAPK/ERK and PI3K/AKT signaling pathways [30–32]. Additionally, GDF15 promotes ErbB1 signal transduction and its tyrosine phosphorylation [33, 34]. Through immunoprecipitation experiments, we found that rhGDF15 treatment on GC cells promoted the binding of ErbB3 to ErbB1 and upregulated the level of ErbB1 phosphorylation (Fig. 5H). Besides, immunoprecipitation experiments also revealed that GDF15 can bind to ErbB1 (Fig. 5I). These suggested that GDF15 can bind and activate ErbB1 through ErbB3 binding and the formation of ErbB3-ErbB1 heterodimer. To verify whether GDF15 activated downstream signaling pathways through ErbB1 activation, we used transwell assays to examine the effect of ErbB1 inhibitor Erlotinib on the migration and invasion of rhGDF15-treated GC cells. The results showed that rhGDF15 promoted migration and invasion of GC cells, which was inhibited upon Erlotinib treatment (Fig. 5J, K). These results indicate that EGR4 could promote the formation of ErbB3-ErbB1 heterodimers through GDF15 to activate the ErbB signaling and downstream MAPK/ERK and PI3K/AKT signaling pathways and promote GC cell motility.

### EGR4 + GC cells activated CAFs through GDF15 to promote ECM remodeling and GC cell motility

**Fig. 6:**
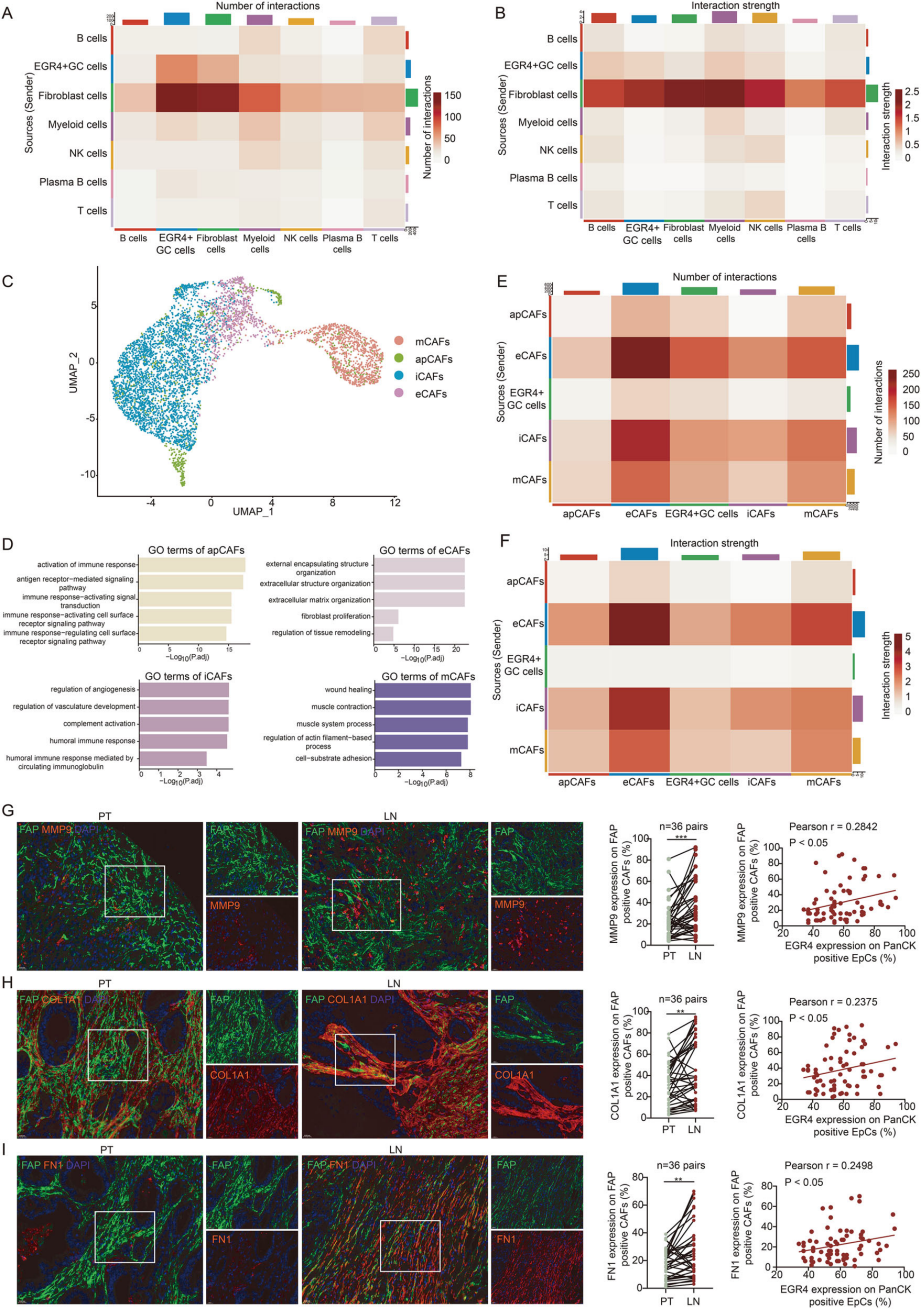
Interaction between EGR4^+^ GC cells and CAFs. **A**, **B** Heatmaps of interactions between EGR4^+^ GC cells and TME cells. **C** UMAP of CAFs showing 4 clusters. **D** GO analysis of the 4 different CAF subtypes. **E**, **F** Heatmaps of interactions between EGR4^+^ GC cells and CAF subtypes. **G–I** Immunofluorescence staining of ECM marker genes in CAFs and correlation analysis with EGR4 in sections of paired primary tumor and lymph node metastasis. FAP, fibroblast activation protein. For each tissue section, three randomly FOVs were analyzed, and the positivity rate of MMP9, COL1A1 or FN1 in FAP-positive CAFs and that of EGR4 in PanCK-positive epithelial cells was calculated for each FOV, with the average value representing the MMP9, COL1A1, FN1 or EGR4 expression level per sample. The paired t-test and Pearson correlation analysis were used for analysis. *p < 0.05, **p < 0.01, ***p < 0.001.

To identify potential interactions between EGR4^+^ GC cells and TME cells, we performed CellChat analysis to map intercellular signaling. The results showed that EGR4^+^ GC cells revealed more and stronger interactions with CAFs (Fig. 6A, B). Through the analysis of TIMER2.0 database, it was found that the survival of patients with high expression of EGR4 and increased infiltration of CAFs in GC was significantly shortened (Figure S6A). Since the complexity of CAFs, we performed unsupervised UMAP clustering analysis and clustered CAFs into four groups (Fig. 6C). Based on GO analysis, these clusters were associated with antigen presentation, extracellular matrix, inflammatory response, and smooth muscle cell-like characteristics, and were thus named apCAF (antigen-presenting CAF), eCAF (extracellular matrix-producing CAF), iCAF (inflammatory CAF), and mCAF (myofibroblastic CAF), respectively (Fig. 6D). Subsequent CellChat analysis revealed that EGR4^+^ GC cells showed more and stronger interactions with eCAFs than other CAF clusters (Fig. 6E, F). Immunofluorescence staining on pairs of metastatic lymph nodes and primary tumors from 36 GC patients showed that the expression of eCAF markers MMP9, COL1A1, and Fibronectin was significantly higher in metastatic lymph nodes than in primary tumors, which was positively correlated with EGR4 level (Fig. 6G–I). These results suggest that the EGR4^+^ GC cells cluster may promote ECM remodeling through interaction and activation of eCAFs to facilitate metastasis.

**Fig. 7:**
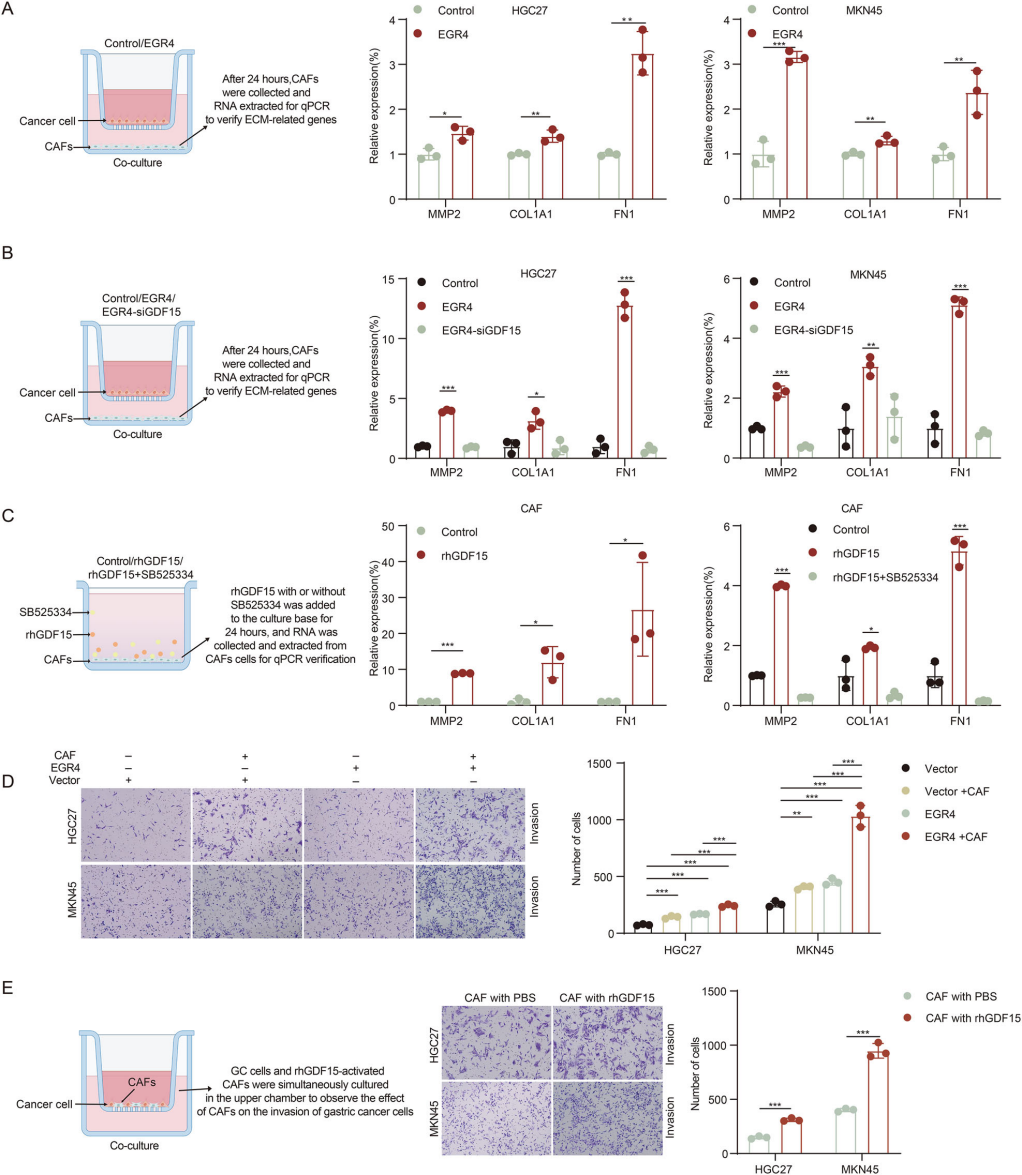
EGR4-upregulated GDF15 in GC cells activated CAFs to promote ECM remodeling and GC cell motility. **A** RT-qPCR detection of ECM remodeling genes in CAFs co-cultured with EGR4 over-expressing or control GC cells. **B** RT-qPCR detection of ECM remodeling genes in CAFs co-cultured with EGR4 over-expressing GC cells upon GDF15 knockdown. **C** RT-qPCR detection of ECM remodeling genes in CAFs treated with rhGDF15 (200 ng/ml) upon treatment of TGFβR inhibitor SB525334 (5 µM). **D** The transwell assay demonstrated the promotion effect of CAFs on the invasion of GC cells. The invaded GC cells were counted, which exhibited significantly smaller cell volumes than the CAFs. **E** The transwell assay demonstrated the role of rhGDF15 (200 ng/ml) treatment on the promotion effect of CAFs on the invasion of GC cells. The invaded GC cells were counted, which exhibited significantly smaller cell volumes than the CAFs. *p < 0.05, **p < 0.01, ***p < 0.001.

To further validate the effect of EGR4^+^ GC cells on eCAFs activation, we co-cultured control and EGR4-overexpressing GC cells with human primary gastric cancer-associated fibroblasts for 24 hours. QPCR experiments showed that co-incubation of EGR4-overexpressing GC cells upregulated genes related to matrix remodeling, such as MMP2, COL1A1 and Fibronectin in CAFs, compared to those incubated with control GC cells (Fig. 7A), which was abolished when GDF15 was knocked down through siRNA (Fig. 7B), suggesting that EGR4 + GC cells promoted eCAFs activation through GDF15. In addition, TIMER2.0 data analysis showed that high expression of GDF15 or EGR4^+^GDF15^+^ and increased CAFs infiltration in gastric cancer patients had a significantly shorter survival time (Figure S6B, C). The TGF-β pathway is a core pathway for CAF activation and ECM remodeling [35–37], and GDF15 could bind to the TGF-β receptor and activates downstream Smad 2/3 pathway [38–40]. To test whether GDF15 promote matrix remodeling through TGF-β receptor, we treated CAFs with rhGDF15 for 24 h, and found that rhGDF15 promoted the CAFs-mediated matrix remodeling process. Besides, TGF-β receptor inhibitor SB525334 treatment significantly impaired the promotion effect of rhGDF15 on matrix remodeling (Fig. 7C). In addition, transwell invasion assay indicated that co-culture with CAFs could promote the invasion of GC cells, which was significantly enhanced in GC cells overexpressing EGR4 (Fig. 7D). Furthermore, rhGDF15 treatment enhanced the promotion effect of CAFs on the invasion of co-cultured GC cells (Fig. 7E). These results suggest that GDF15 secreted by EGR4^+^ GC cells could activate CAF-mediated matrix remodeling through the TGF-β pathway to promote cell motility.

## Discussion

Gastric cancer (GC) is a highly heterogeneous malignant tumor, and despite the widespread application of scRNA-seq in resolving its heterogeneity, the critical tumor cell subpopulations driving GC metastasis and their underlying molecular mechanisms remain poorly understood [41, 42]. In this study, we employed scRNA-seq to analyze the metastatic lymph nodes and paired primary tumors from GC patients, leading to the identification of an EGR4+ cancer cell subpopulation strongly associated with GC metastasis. This subpopulation was demonstrated to play a pivotal role in metastasis, highlighting its clinical relevance.

Our findings demonstrate that EGR4 directly binds to the promoter region of *GDF15*, upregulating its expression. GDF15, in turn, interacts with ErbB3, enhancing the interaction between ErbB3 and ErbB1 and activating the ErbB1 signaling pathway. ErbB1 inhibitor Erlotinib effectively suppressed the migration and invasion of rhGDF15-treated GC cells. These results suggest that the GDF15-ErbB3/ErbB1 axis may be a potential therapeutic target for intervening GC metastasis. However, the precise mechanism by which GDF15 enhances the interaction between ErbB3 and ErbB1 remains to be elucidated and warrants further investigation.

Additionally, we uncover that GDF15 promotes metastasis by activating the TGF-β signaling pathway in ECM-producing eCAFs. This activation induces eCAFs to secrete ECM components (e.g., collagen, fibronectin) and proteases (e.g., matrix metalloproteinases, MMPs), which remodel the TME into a “stiffened” matrix. This remodeling provides physical support for cancer cells and activates mechanosensitive pathways such as YAP/TAZ, thereby enhancing cancer cell migration and invasion [43]. Furthermore, eCAF-derived growth factors (e.g., TGF-β, VEGF) induce epithelial-mesenchymal transition to further facilitate metastasis [44, 45]. Given the diversity of GDF15 receptors and their expression patterns, future studies should explore the role of the EGR4/GDF15 axis in immune regulation during GC metastasis.

Our findings have significant translational implications for the precision treatment of GC metastasis. Targeting the EGR4/GDF15 axis may offer a novel therapeutic strategy. For instance, the development of small-molecule inhibitors specifically targeting EGR4 could suppress GDF15 expression and inhibit metastasis. Neutralizing antibodies against GDF15 or soluble ErbB3 receptor traps could block the interaction between GDF15 and ErbB3 [46], effectively inhibit the metastatic potential of EGR4^+^ GC cells. Additionally, TGF-β receptor inhibitors (e.g., SB525334) and MMP inhibitors (e.g., Marimastat) could attenuate GDF15-induced eCAF activation, thereby reducing the supportive role of the TME in GC metastasis. Combination therapies involving ErbB1 inhibitors (e.g., Erlotinib) and GDF15-neutralizing antibodies may also yield synergistic effects.

In conclusion, the present study identifies the EGR4 + GC cell subpopulation as a critical driver of GC metastasis and elucidates the molecular mechanisms by which the EGR4/GDF15 axis promotes GC metastasis through the activation of the ErbB3/ErbB1 signaling and eCAF-mediated ECM remodeling. These findings not only provide new insights into the molecular mechanisms underlying GC metastasis but also lay the groundwork for the development of targeted therapies against the EGR4/GDF15 axis.

## Supplementary information

supplementary figures

FigureS1

FigureS2

FigureS3

FigureS4

FigureS5

FigureS6

Supplemental tables

checklist

Original Western blots

Western Blots Statistical Test Chart

## Data Availability

All raw data are in the Genome Sequence Archive for Human (GSA-Human) at the National Genomics Data Center under the accession number HRA009590 (http://bigd.big.ac.cn/gsa-human).
