# Supplementary material for: EGR4 transcriptionally upregulates GDF15 to promote gastric cancer metastasis
Source: Cell Death Dis. 2025 Nov 7;16(1):807. doi: 10.1038/s41419-025-08095-w (PMC12594975; doi:10.1038/s41419-025-08095-w)
Supplement: Supplementary file 1 — supplementary figures [file 41419_2025_8095_MOESM1_ESM.docx]

**Supplementary Figures**

**Figure S1: Expression profiling of 59,918 single cells from primary tumors and metastatic lymph nodes of GC patients.**

(**A**) UMAP plots of 59918 cells from primary tumor and paired lymph node tissue of six GC patients, showing 19 clusters in each plot. (B) Heatmap showing the expression of top marker genes from each cluster relative to Figure 1A. (C) Expression levels of selected marker genes across 59918 unsorted cells illustrated in UMAP plots from both primary cancer and lymph node tissue of GC patients.

**Figure S2: Kaplan-Meier survival and tumor progression analysis based on expression of genes in C2 clusters.**

(**A**) Kaplan-Meier survival curves showing significant differences in overall survival between GC patients with higher and lower KRT20, REG1B, MUC13, PRAP1, SPINK4, REG4 and REG1A expression. (**B-C**) Kaplan-Meier plots illustrating significant differences in tumor progression between GC patients with higher and lower KRT20, REG1B, MUC13, PRAP1, SPINK4, REG4 and REG1A expression. *P* values were computed using Kaplan-Meier analysis.

**Figure S3: Kaplan-Meier survival and tumor progression analysis based on expression levels of genes in C9 clusters.**

(**A**) Kaplan-Meier survival curves showing significant differences in overall survival between GC patients with higher and lower SNAP25, RAB3IP3, KCNB2, CHGA, NPAS3, PALLD, CPE, GHRL and CACNB2 expression. (**B-C**) Kaplan-Meier plots illustrating significant differences in tumor progression between GC patients with higher and lower SNAP25, RAB3IP3, KCNB2, CHGA, NPAS3, PALLD, CPE, GHRL and CACNB2 expression. *P* values were computed using Kaplan-Meier analysis.

**Figure S4: Kaplan-Meier analysis based on NEUROD1 and NEUROD2 expression in GC patients.**

1. **C**) Kaplan-Meier survival curves showing significant differences in overall survival and tumor progression between GC patients with higher and lower NEUROD1 expression. (**D-F**) Kaplan-Meier survival curves showing significant differences in overall survival and tumor progression between GC patients with higher and lower NEUROD2 expression. *P* values were computed using Kaplan-Meier analysis.

**Figure S5: Statistical graph of immunostaining for EGR4, GDF15, p-PI3K and p-ERK.**

(**A**) The number of EGR4^+^GDF15^+^p-PI3K^+^ and EGR4^+^GDF15^+^p-ERK^+^ cells in the tissue sections from the control group and the EGR4 overexpression group.***p<0.001by unpaired t-test. (**B**) The number of EGR4^+^GDF15^+^p-PI3K^+^ and EGR4^+^GDF15^+^p-ERK^+^ cells in the tissue sections from the negative control group and the EGR4 knockdown group. Fifteen fields of the slides per group were counted and statistically analyzed. ***p<0.001by unpaired t-test.*, *p*<0.05; **, *p*<0.01; ***, *p*<0.001.

**Figure S6: Kaplan-Meier analysis based on EGR4,GDF15 and EGR4^+^GDF15^+^ expression in GC patients.**

(**A**) Kaplan-Meier survival analysis of GC patients with different EGR4 expression levels stratified by levels of infiltrating CAFs. (**B**) Kaplan-Meier survival analysis of GC patients with different GDF15 expression levels stratified by levels of infiltrating CAFs. (**C**) Kaplan-Meier survival analysis of GC patients with different EGR4^+^GDF15^+^ expression levels stratified by levels of infiltrating CAFs.
