# Supplementary figures and images for: EGR4 transcriptionally upregulates GDF15 to promote gastric cancer metastasis
Source: Cell Death Dis. 2025 Nov 7;16(1):807. doi: 10.1038/s41419-025-08095-w (PMC12594975; doi:10.1038/s41419-025-08095-w)

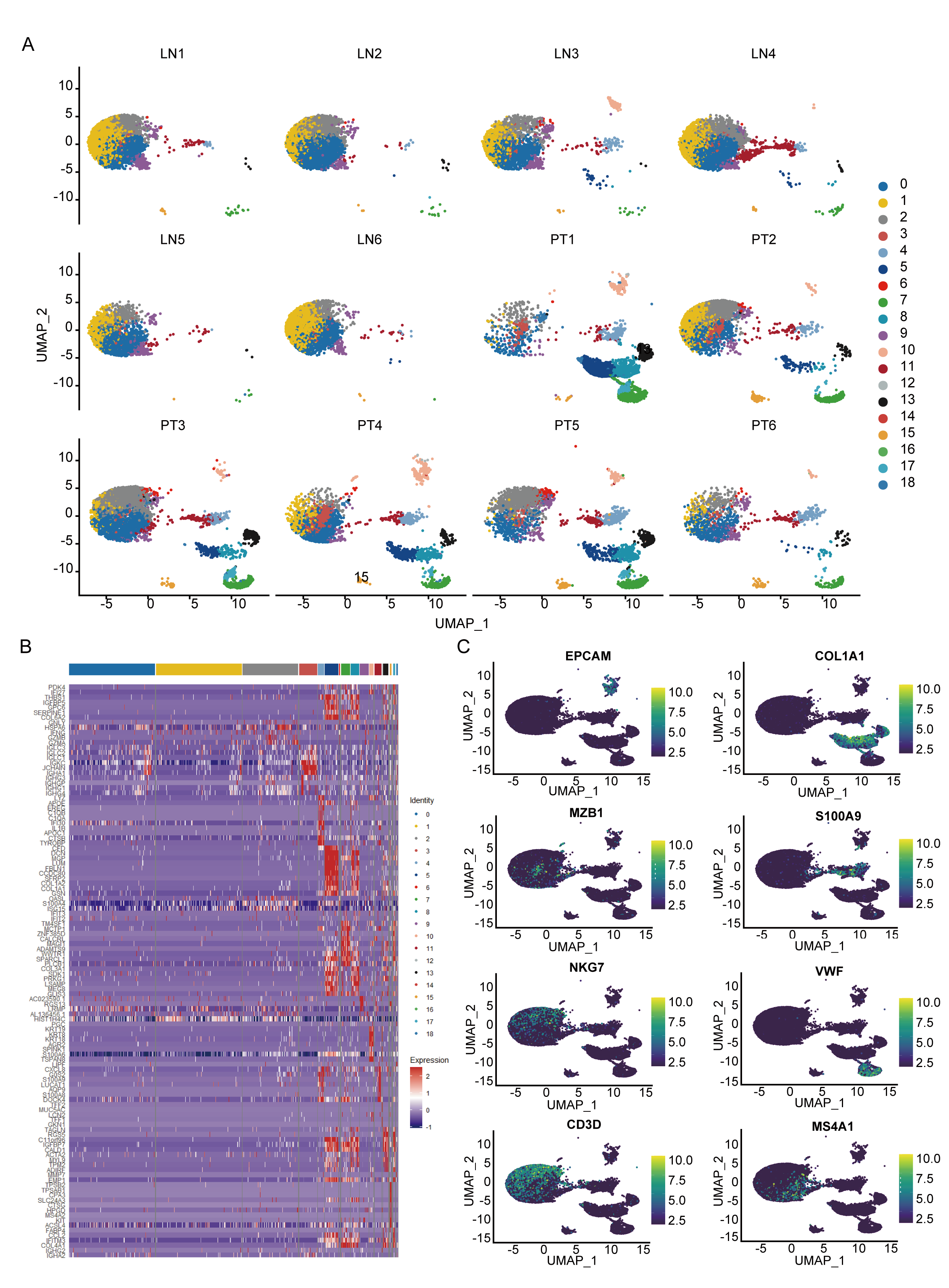

Supplement: Supplementary file 2 — FigureS1 [file 41419_2025_8095_MOESM2_ESM.png]

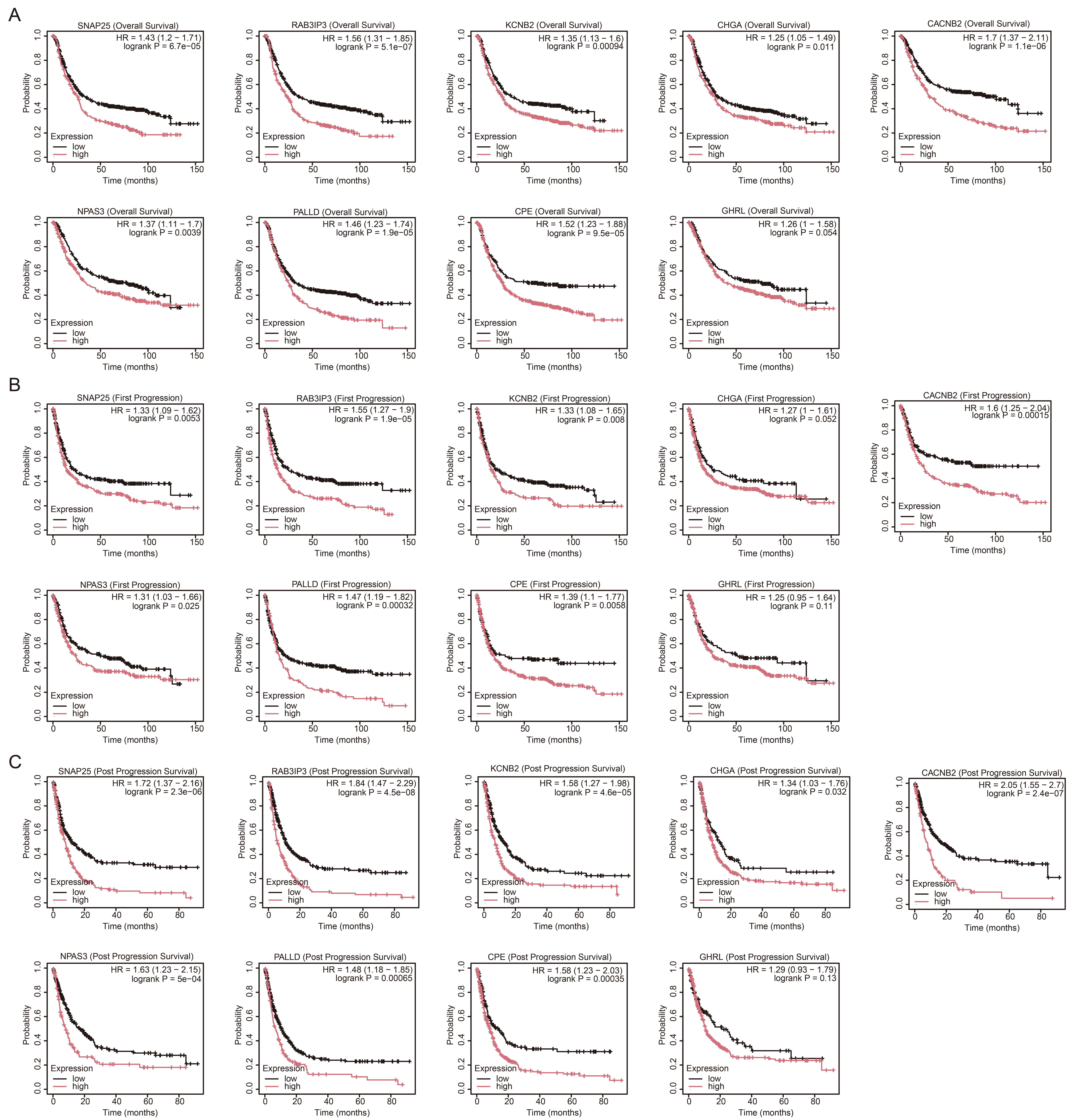

Supplement: Supplementary file 4 — FigureS3 [file 41419_2025_8095_MOESM4_ESM.png]

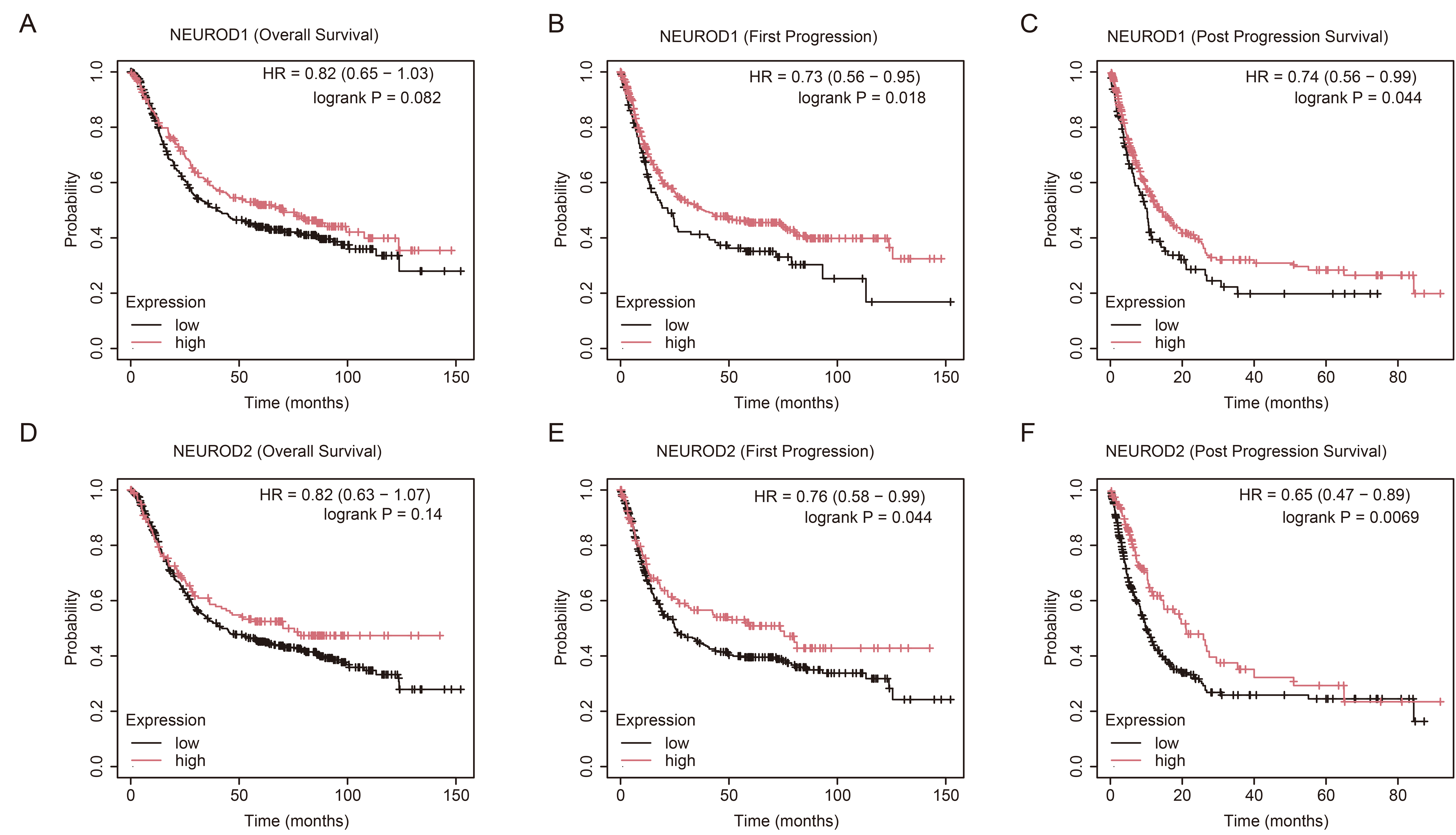

Supplement: Supplementary file 5 — FigureS4 [file 41419_2025_8095_MOESM5_ESM.png]

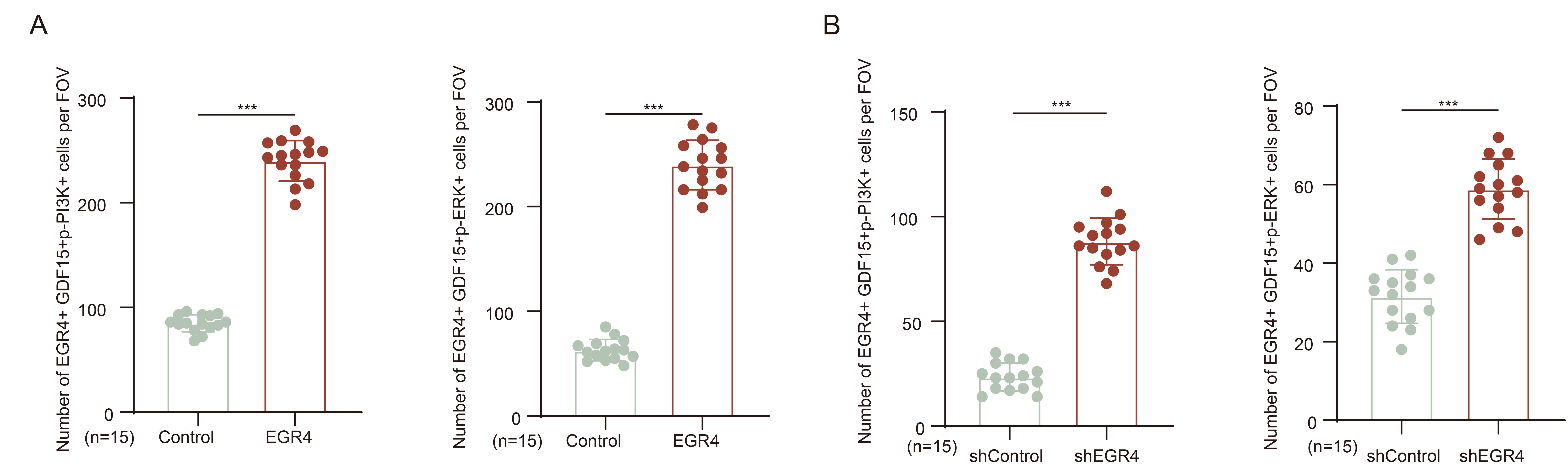

Supplement: Supplementary file 6 — FigureS5 [file 41419_2025_8095_MOESM6_ESM.png]

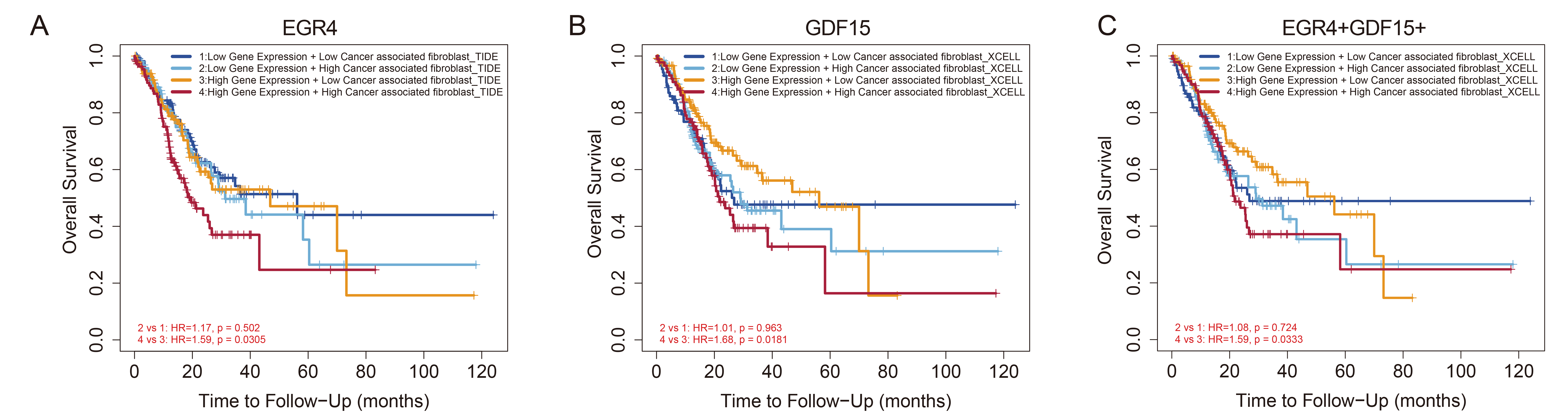

Supplement: Supplementary file 7 — FigureS6 [file 41419_2025_8095_MOESM7_ESM.png]

2H

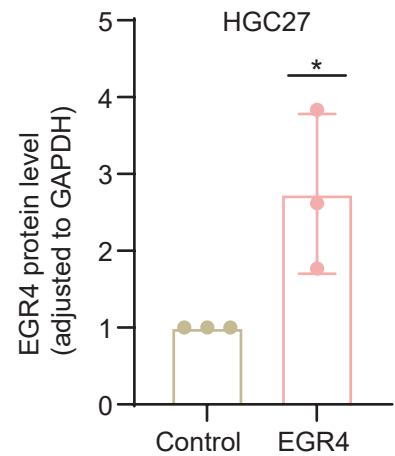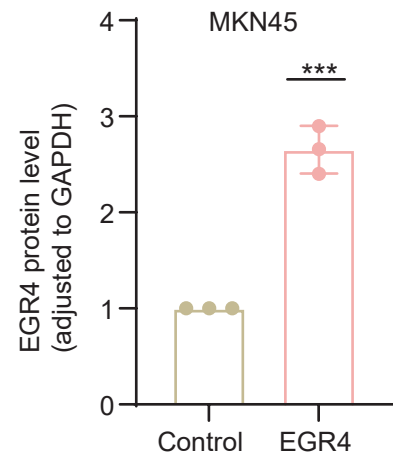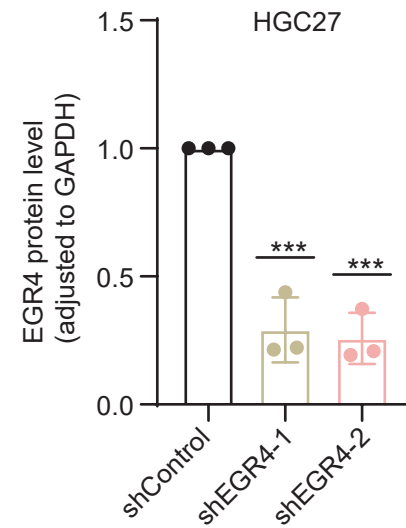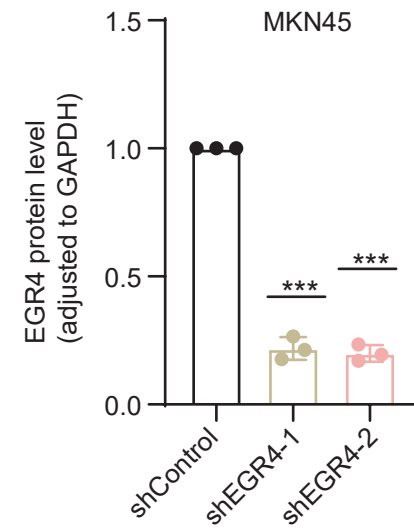

4K

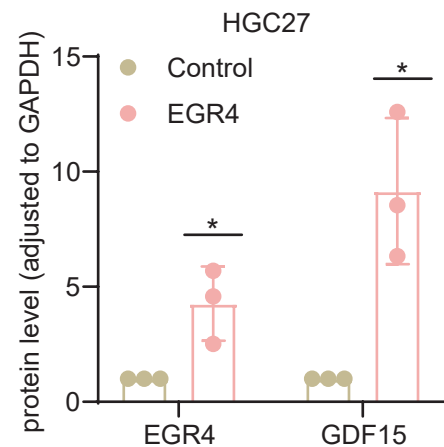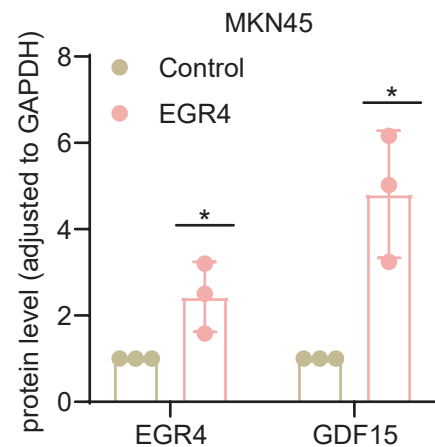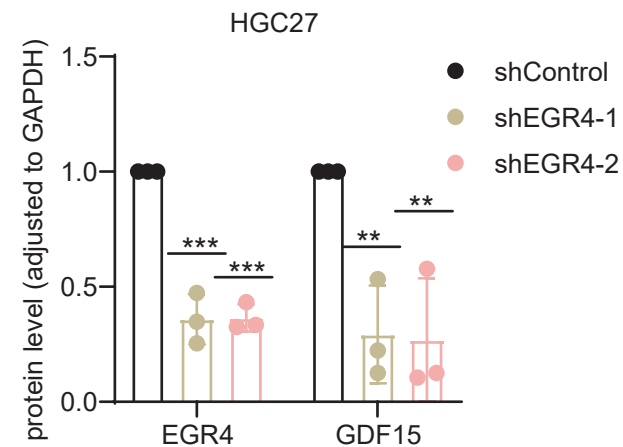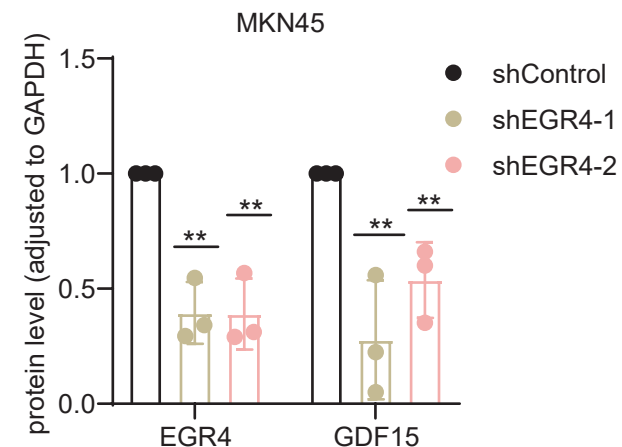

5A

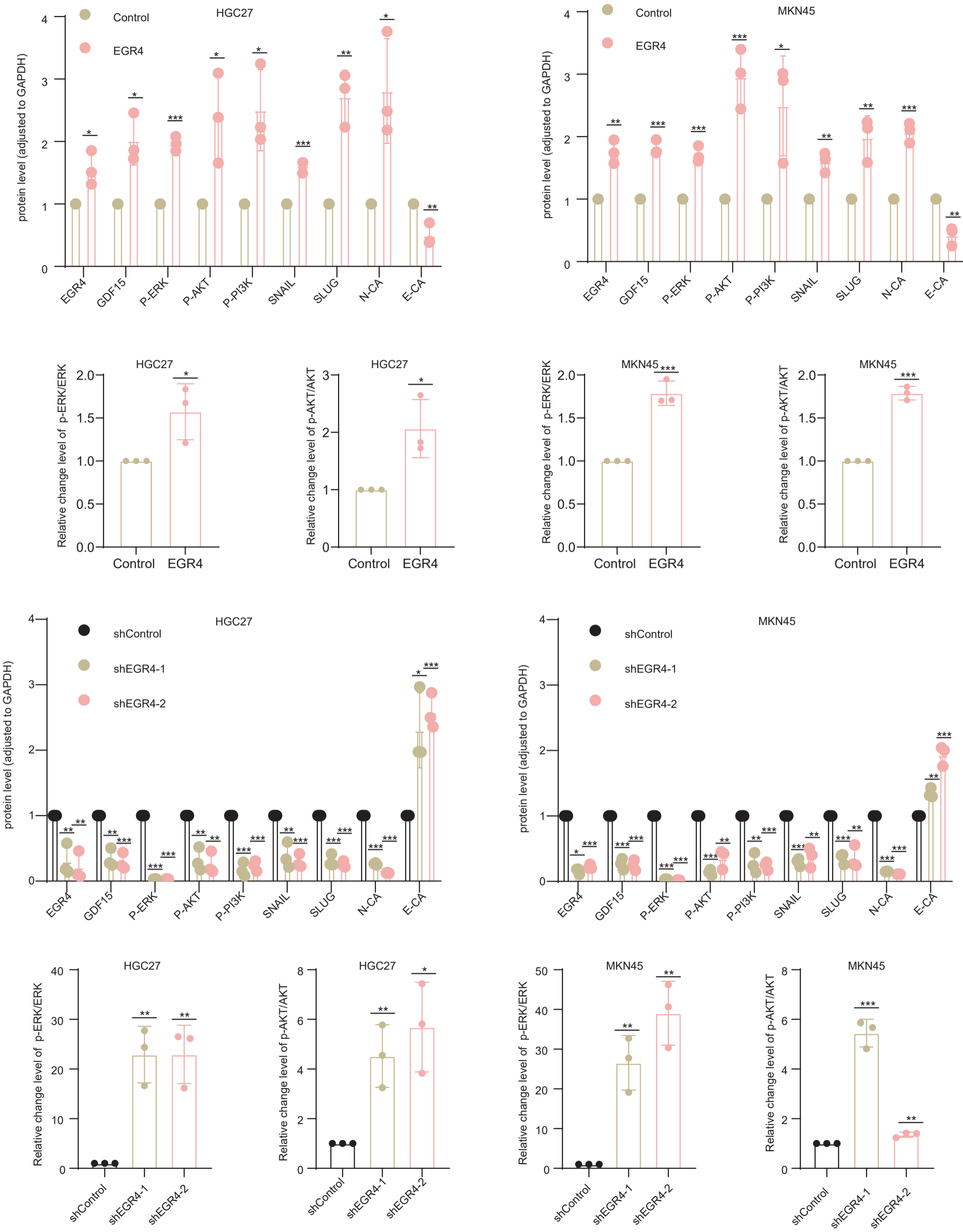

5C

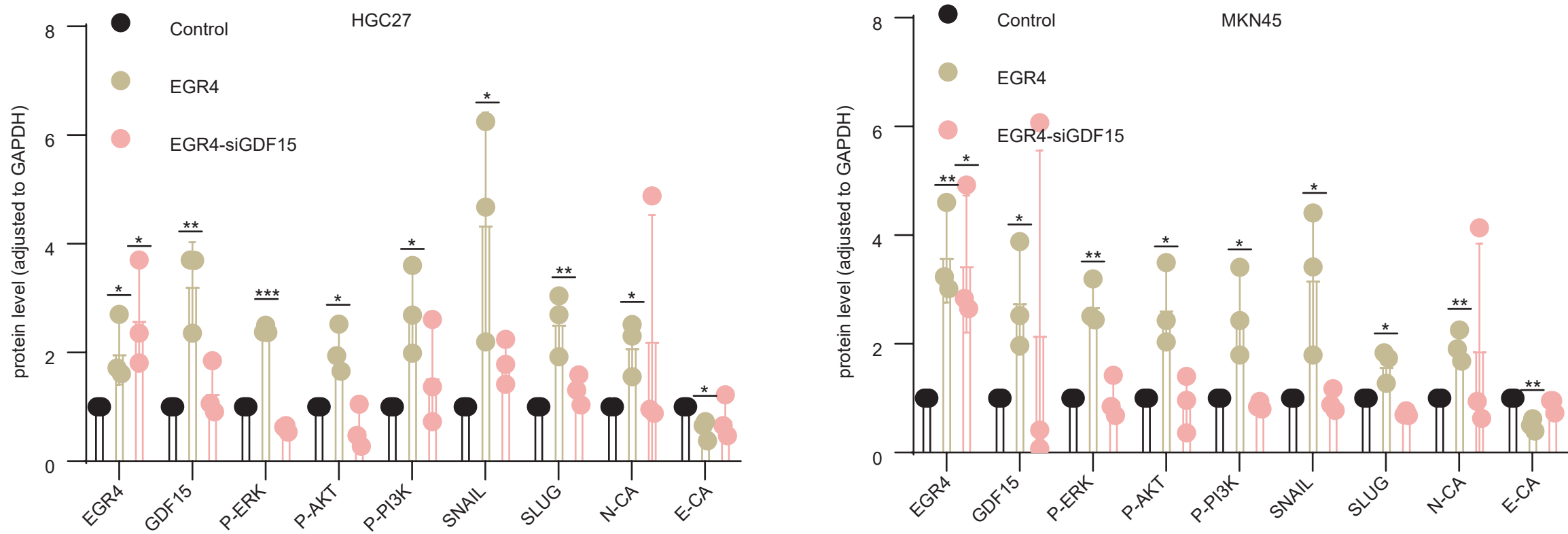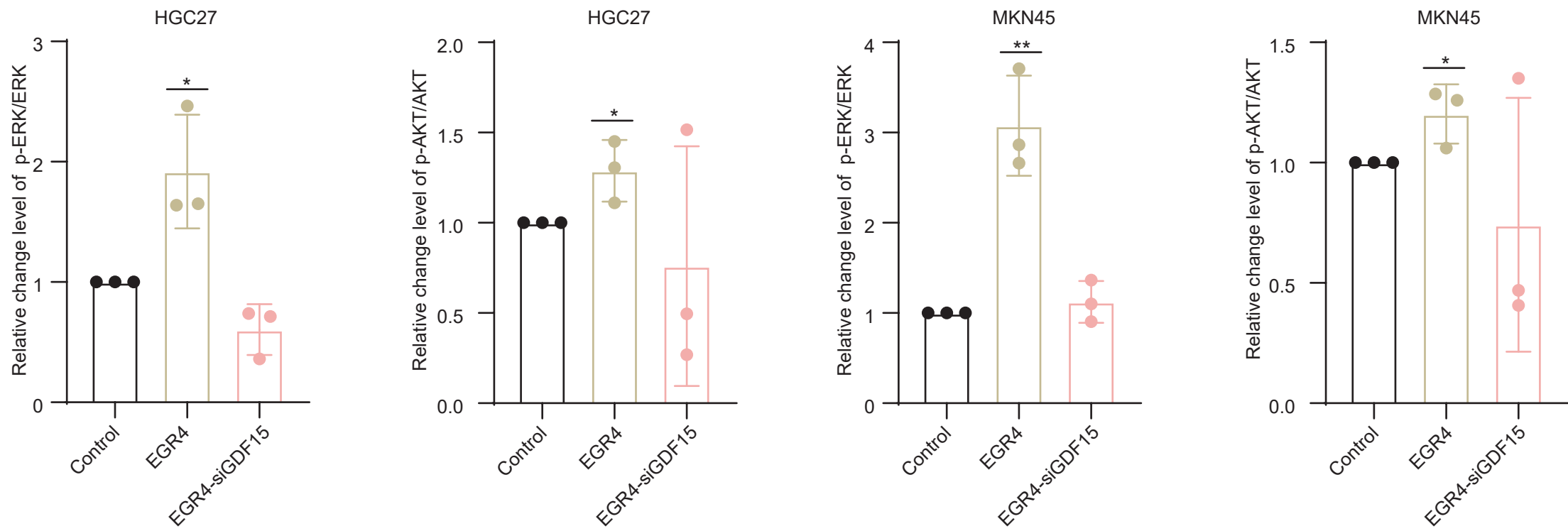

5H

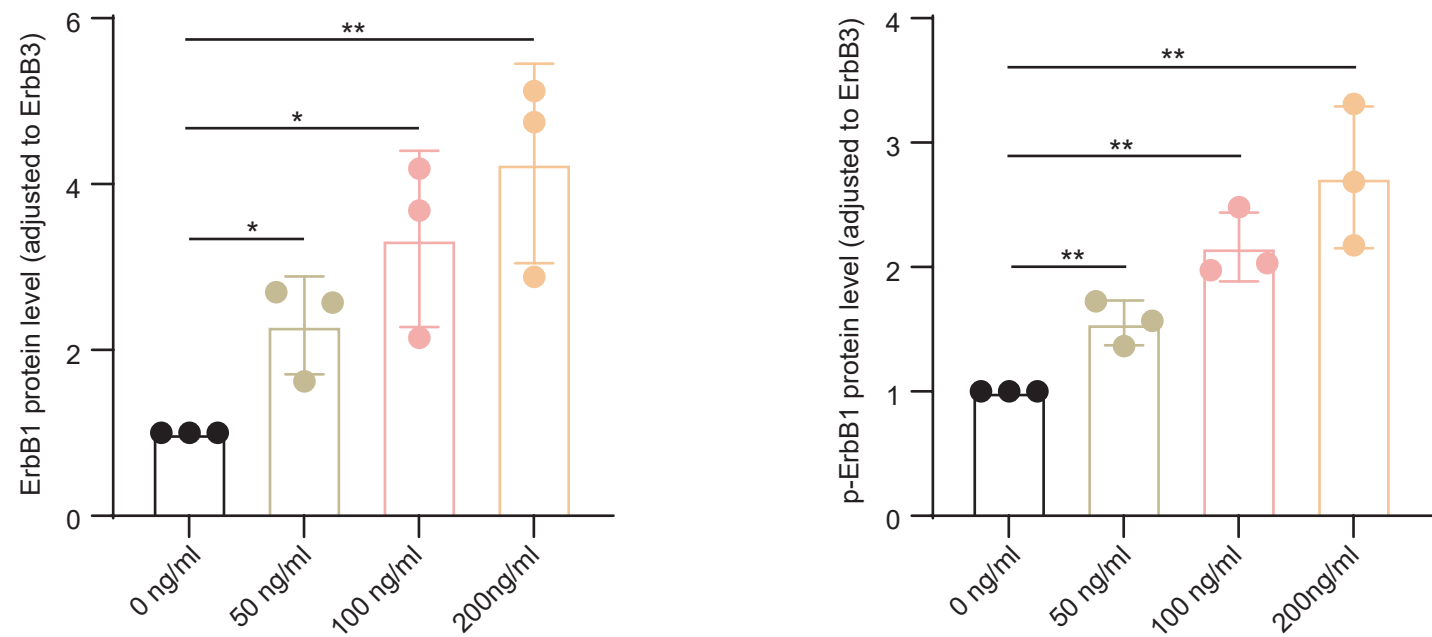

Supplement: Supplementary file 11 — Western Blots Statistical Test Chart [file 41419_2025_8095_MOESM11_ESM.pdf]
