# Supplementary material for: EGR4 transcriptionally upregulates GDF15 to promote gastric cancer metastasis
Source: Cell Death Dis. 2025 Nov 7;16(1):807. doi: 10.1038/s41419-025-08095-w (PMC12594975; doi:10.1038/s41419-025-08095-w)
Supplement: Supplementary file 8 — Supplemental tables [file 41419_2025_8095_MOESM8_ESM.docx]

| Number of cases | name | TNM staging | Tumor staging | Early stage; Later period |
| --- | --- | --- | --- | --- |
| **1** | PT1 | T3N1 | IIB | Later period |
| **2** | LN1 |  |  |  |
| **3** | PT2 | T1bN0 | IA | Later period |
| **4** | LN2 |  |  |  |
| **5** | PT3 | T4aN3b | IIIC | Early stage |
| **6** | LN3 |  |  |  |
| **7** | PT4 | T3N3a | IIIB | Later period |
| **8** | LN4 |  |  |  |
| **9** | PT5 | T2N0 | IB | Later period |
| **10** | LN5 |  |  |  |
| **11** | PT6 | T3N2 | IIA | Later period |
| **12** | LN6 |  |  |  |

**Supplemental Table1. The clinicopathological characteristics of the patients in scRNA-seq.**

**Supplemental Table2. The clinicopathological characteristics of the patients in TMA.**

| **Location** | **Age** | **Gender** | **Classification of Pathology** | **Grade of pathology** | **TNM** |
| --- | --- | --- | --- | --- | --- |
| C1 | 54 | M | Adenocarcinoma Carcinoma Lymph node metastasis | IIB | T3N1M0 |
| D1 |  |  |  |  |  |
| C2 | 60 | M | Adenocarcinoma Carcinoma Lymph node metastasis | IIA | T2N1M0 |
| D2 |  |  |  |  |  |
| C3 | 42 | M | Adenocarcinoma Carcinoma Lymph node metastasis | IIIA | T3N2M0 |
| D3 |  |  |  |  |  |
| C4 | 59 | F | Adenocarcinoma Carcinoma Lymph node metastasis | IIIA | T3N2M0 |
| D4 |  |  |  |  |  |
| C5 | 50 | M | Adenocarcinoma Carcinoma Lymph node metastasis | IIIA | T3N2M0 |
| D5 |  |  |  |  |  |
| C6 | 52 | F | Signet ring cell Carcinoma Lymph node metastasis | IIB | T3N1M0 |
| D6 |  |  |  |  |  |
| C7 | 68 | F | Adenocarcinoma Carcinoma Lymph node metastasis | IIIA | T3N2M0 |
| D7 |  |  |  |  |  |
| C8 | 63 | M | Adenocarcinoma Carcinoma Lymph node metastasis | IIIA | T3N2M0 |
| D8 |  |  |  |  |  |
| C9 | 61 | M | Adenocarcinoma Carcinoma Lymph node metastasis | IIIA | T3N2M0 |
| D9 |  |  |  |  |  |
| C10 | 56 | M | Signet ring cell Carcinoma Lymph node metastasis | IIIA | T3N2M0 |
| D10 |  |  |  |  |  |
| C11 | 77 | M | Undifferentiated Carcinoma Lymph node metastasis | IIIA | T3N2M0 |
| D11 |  |  |  |  |  |
| C12 | 80 | F | Adenocarcinoma Carcinoma Lymph node metastasis | IIIA | T3N2M0 |
| D12 |  |  |  |  |  |
| E1 | 38 | F | Undifferentiated Carcinoma Lymph node metastasis | IIB | T2N2M0 |
| F1 |  |  |  |  |  |
| E2 | 40 | F | Adenocarcinoma Carcinoma Lymph node metastasis | IIB | T3N1M0 |
| F2 |  |  |  |  |  |
| E3 | 45 | F | Adenocarcinoma Carcinoma Lymph node metastasis | IIB | T3N1M0 |
| F3 |  |  |  |  |  |
| E4 | 70 | M | Adenocarcinoma Carcinoma Lymph node metastasis | IIIA | T3N2M0 |
| F4 |  |  |  |  |  |
| E5 | 68 | M | Adenocarcinoma Carcinoma Lymph node metastasis | IIIA | T3N2M0 |
| F5 |  |  |  |  |  |
| E6 | 33 | F | Undifferentiated Carcinoma Lymph node metastasis | IIIA | T3N2M0 |
| F6 |  |  |  |  |  |
| E7 | 60 | M | Adenocarcinoma Carcinoma Lymph node metastasis | IIIA | T3N2M0 |
| F7 |  |  |  |  |  |
| E8 | 45 | M | Adenocarcinoma Carcinoma Lymph node metastasis | IIIA | T3N2M0 |
| F8 |  |  |  |  |  |
| E9 | 73 | M | Adenocarcinoma Carcinoma Lymph node metastasis | IIB | T3N1M0 |
| F9 |  |  |  |  |  |
| E10 | 57 | M | Signet ring cell Carcinoma Lymph node metastasis | IIIA | T3N2M0 |
| F10 |  |  |  |  |  |
| E11 | 31 | F | Adenocarcinoma Carcinoma Lymph node metastasis | IIIA | T3N2M0 |
| F11 |  |  |  |  |  |
| E12 | 54 | M | Adenocarcinoma Carcinoma Lymph node metastasis | IIB | T3N1M0 |
| F12 |  |  |  |  |  |
| G1 | 62 | M | Adenocarcinoma Carcinoma Lymph node metastasis | IIIA | T3N2M0 |
| H1 |  |  |  |  |  |
| G2 | 64 | F | Adenocarcinoma Carcinoma Lymph node metastasis | IIIA | T3N2M0 |
| H2 |  |  |  |  |  |
| G3 | 51 | M | Signet ring cell Carcinoma Lymph node metastasis | IIIA | T3N2M0 |
| H3 |  |  |  |  |  |
| G4 | 61 | M | Adenocarcinoma Carcinoma Lymph node metastasis | IIB | T3N1M0 |
| H4 |  |  |  |  |  |
| G5 | 57 | F | Adenocarcinoma Carcinoma Lymph node metastasis | IIIA | T3N2M0 |
| H5 |  |  |  |  |  |
| G6 | 60 | M | Adenocarcinoma Carcinoma Lymph node metastasis | IIB | T3N1M0 |
| H6 |  |  |  |  |  |
| G7 | 40 | F | Adenocarcinoma Carcinoma Lymph node metastasis | IIIA | T3N2M0 |
| H7 |  |  |  |  |  |
| G8 | 57 | M | Adenocarcinoma Carcinoma Lymph node metastasis | IIIA | T3N2M0 |
| H8 |  |  |  |  |  |
| G9 | 64 | M | Adenocarcinoma Carcinoma Lymph node metastasis | IIIA | T4N1M0 |
| H9 |  |  |  |  |  |
| G10 | 47 | M | Adenocarcinoma Carcinoma Lymph node metastasis | IIA | T1N2M0 |
| H10 |  |  |  |  |  |
| G11 | 68 | F | Signet ring cell Carcinoma Lymph node metastasis | IIB | T3N1M0 |
| H11 |  |  |  |  |  |
| G12 | 60 | M | Adenocarcinoma Carcinoma Lymph node metastasis | IIIA | T3N2M0 |
| H12 |  |  |  |  |  |

**Supplemental Table3. The antibody information.**

| Antibody | Cat NO. | Company |
| --- | --- | --- |
| Panck | C2562 | Sigma |
| SNAP25 | F1963 | Selleck |
| EGR4 | ab198197 | Abcam |
| GAPDH | 10494-1-AP | Proteintech |
| GDF15 | F1086 | Selleck |
| ERK | T40071 | Abmart |
| p-ERK | T40072 | Abmart |
| AKT | T55561 | Abmart |
| p-AKT | T40067 | Abmart |
| PI3K | [T40115](http://www.ab-mart.com.cn/page.aspx?node=%2077%20&id=%2049665) | Abmart |
| p-PI3K | T40116 | Abmart |
| Snail1 | [TA6032](http://www.ab-mart.com.cn/page.aspx?node=%2077%20&id=%2020225) | Abmart |
| Slug | [TA6032](http://www.ab-mart.com.cn/page.aspx?node=%2077%20&id=%2020225) | Abmart |
| N-cadherin | [T55015](http://www.ab-mart.com.cn/page.aspx?node=%2077%20&id=%201319) | Abmart |
| E-cadherin | [TA0131](http://www.ab-mart.com.cn/page.aspx?node=%2077%20&id=%2022176) | Abmart |
| Fibronectin | T59537 | Abmart |
| Vimentin | T55134 | Abmart |
| ErbB1 | bsm-52317R | Bioss |
| p-ErbB1 | F0298 | Selleck |
| ErbB3 | F0433 | Selleck |
| FAP | ab314456 | Abcam |
| MMP9 | ABB5205 | HuiLanBio |
| COL3A1 | HL22517 | HuiLanBio |
| Fibronectin | Ab2413 | Abcam |
| FLAG | ab205606 | Abcam |

**Supplemental Table4. The sequences of primers.**

| Primer | Sequence (5’ to 3’) |
| --- | --- |
| EGR4-F | CAGCGACC ACCTCACCA |
| EGR4-R | CTGTGCCGTTTCTTCTCGT |
| KCNB2-F | CCTGGCGCTGCGGCTTTGTCC |
| KCNB2-R | AGGCGTGTCCGGGGCAGTCG |
| CHGA-F | GAATAAAGGGGACACTGAGGTGAT |
| CHGA-R | TCCTCGGAGCGTCTCAAAAC |
| RIMS2-F | GGTTCGGCTCCACCAAACAT |
| RIMS2-R | TTTCCTCTCCTCCTCCGTGA |
| GDF15-F | GACCCTCAGAGTTGCACTCC |
| GDF15-R | GCCTGGTTAGCAGGTCCTC |
| TRIB3-F | AAGCGGTTGGAGTTGGATGAC |
| TRIB3-R | CACGATCTGGAGCAGTAGGTG |
| SNAP25-F | ACCAGTTGGCTGATGAGTCG |
| SNAP25-R | CAAAGTCCTGATACCAGCATCTT |
| GHRL-F | TACTACTCTCCACGCCC |
| GHRL-R | AGGGGCCATCCACAGTCTTC |
| NPAS3-F | TGTCTTTGACTATGTCCACCCC |
| NPAS3-R | ACACTGTAATGCCTTCGCCC |
| Pallad-F | AACCGAGCAGGACAGAAC |
| Pallad-R | TGGTGGCACTCCCAATAC |
| MMP2-F | CGACCACAGCCAACTACGATGATG |
| MMP2-R | GTGCCAAGGTCAATGTCAGGAGAG |
| Col1a1-F | CGCAAAGAGTCTACATGTCTAGG |
| Col1a1-R | CATTGTGTATGCAGCTGACTTC |
| Fn1-F | GAGCTATCCATTTCACCTTCAGA |
| Fn1-R | TTGTTCGTAGACACTGGAGAC |

**Supplemental Table5. The sequences of shRNA and siRNA targets.**

| shRNA/siRNA | Sequence (5’ to 3’) |
| --- | --- |
| EGR4-sh1 | GGACCAAGATTGAGGACTT |
| EGR4-sh2 | GCTACAGCGGTAGCTTCTT |
| siGDF15-1 | CTCAGAGTTGCACTCCGAA |
| siGDF15-2 | GGATACTCACGCCAGAAGT |
| siGDF15-3 | TCAGATGCTCCTGGTGTTG |
