# Supplementary material for: EGR4 transcriptionally upregulates GDF15 to promote gastric cancer metastasis
Source: Cell Death Dis. 2025 Nov 7;16(1):807. doi: 10.1038/s41419-025-08095-w (PMC12594975; doi:10.1038/s41419-025-08095-w)
Supplement: Supplementary file 10 — Original Western blots [file 41419_2025_8095_MOESM10_ESM.pdf]

figure2

2H

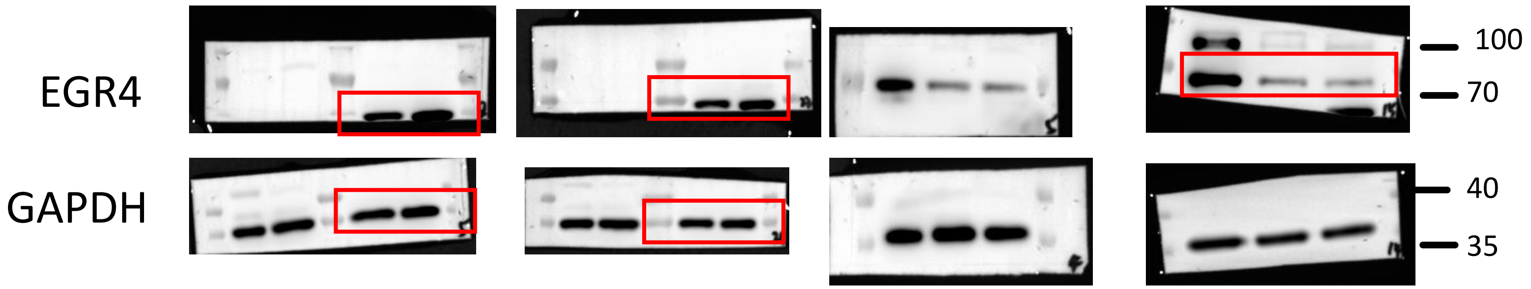

figure4

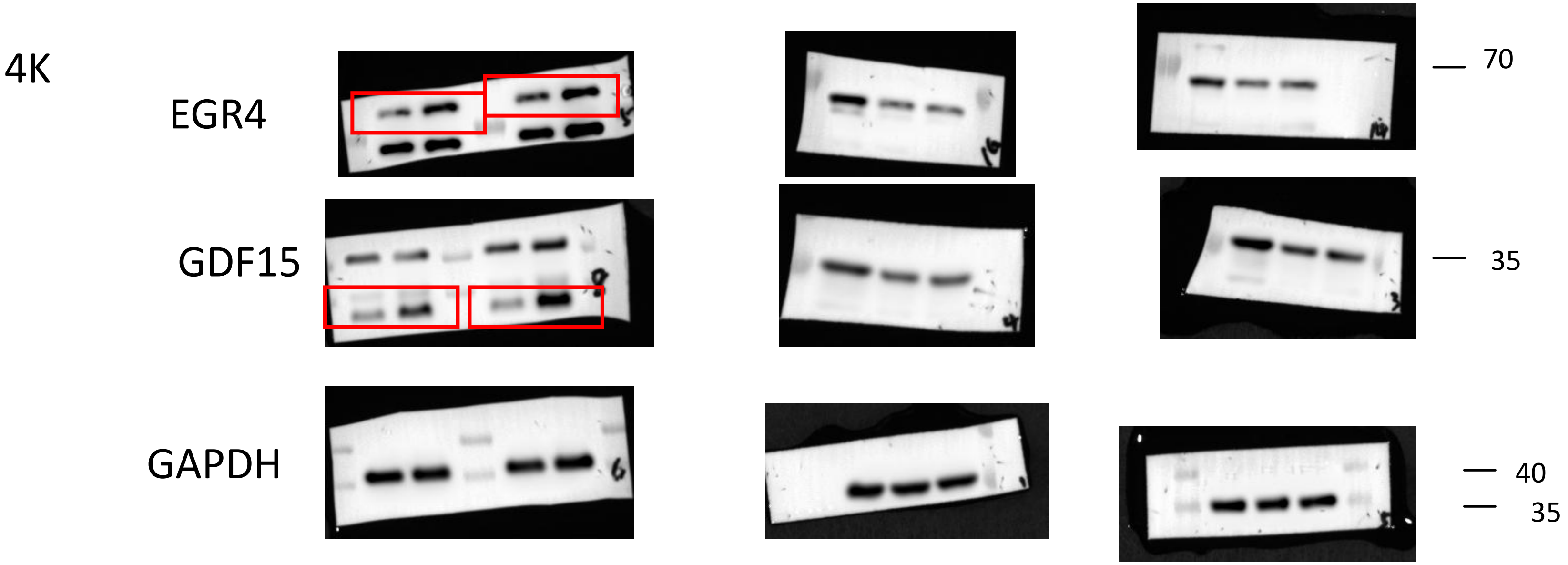

figure5

5A

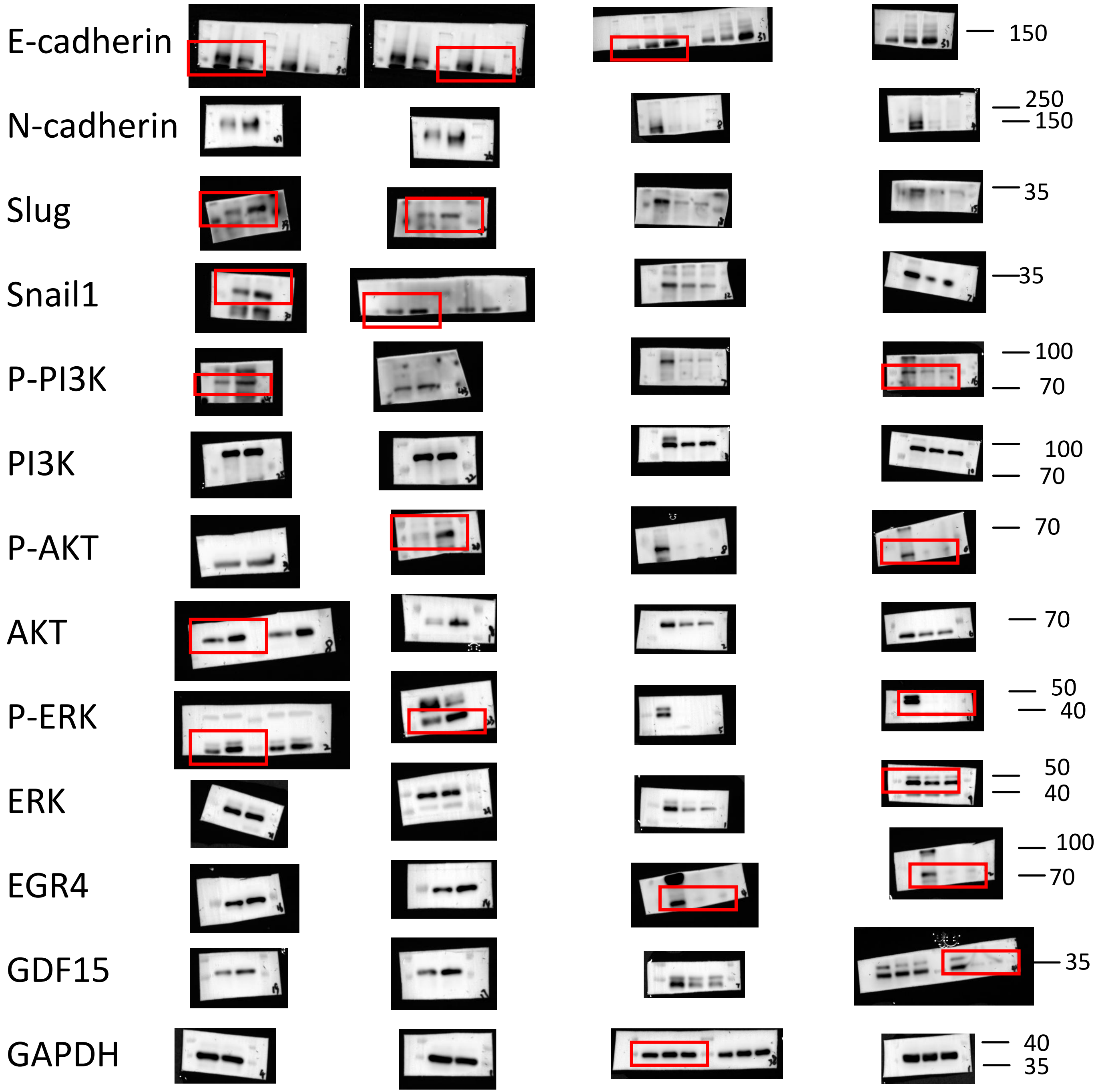
E-cadherin
N-cadherin
Slug
Snail1
P-PI3K
PI3K
P-AKT
AKT
P-ERK
ERK
EGR4
GDF15
GAPDH
150
250
150
35
35
100
70
100
70
70
70
50
40
50
40
100
70
35
40
35

figure5

5C

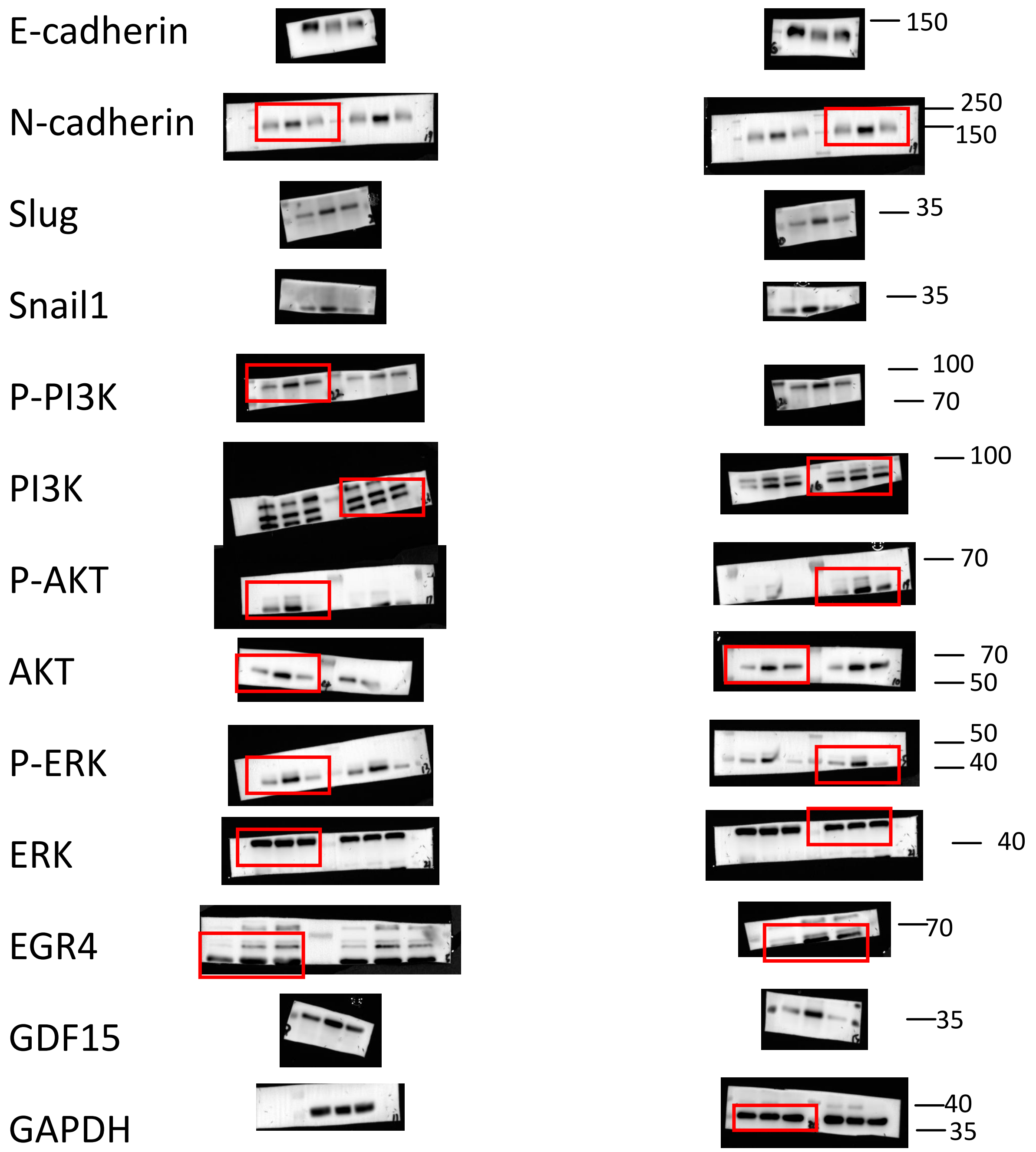
E-cadherin
N-cadherin
Slug
Snail1
P-PI3K
PI3K
P-AKT
AKT
P-ERK
ERK
EGR4
GDF15
GAPDH
150
250
150
35
35
100
70
100
70
70
50
50
40
40
70
35
40
35

figure5

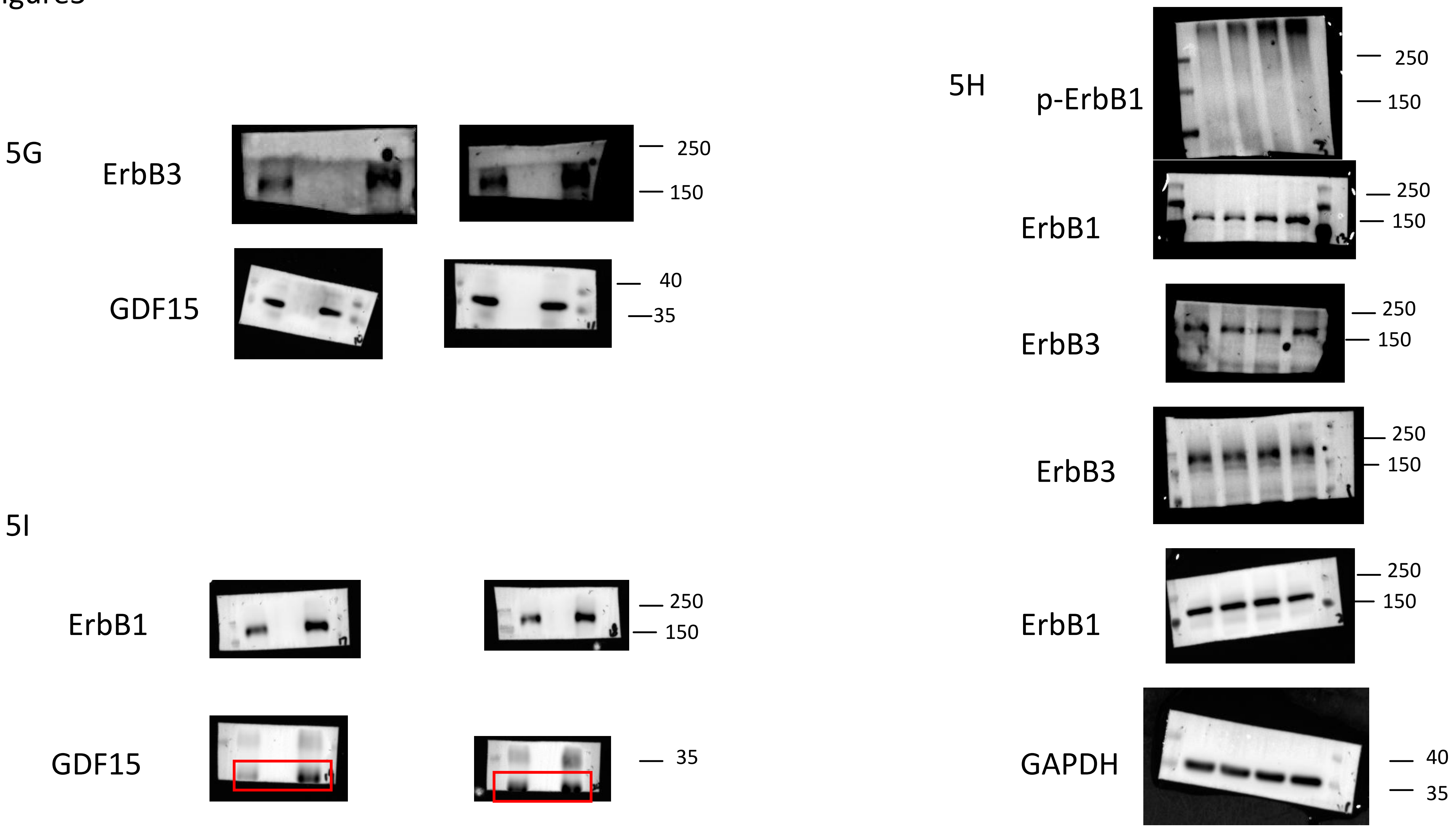

5G
ErbB3
250
150
GDF15
40
35
5I
ErbB1
250
150
GDF15
35
5H
p-ErbB1
250
150
ErbB1
250
150
ErbB3
250
150
ErbB3
250
150
ErbB1
250
150
GAPDH
40
35
